# The Association of Suppressed Hypoxia-Inducible Factor-1 Transactivation of Angiogenesis With Defective Recovery From Cerebral Ischemic Injury in Aged Rats

**DOI:** 10.3389/fnagi.2021.648115

**Published:** 2021-02-26

**Authors:** Yingjia Guo, Junpeng Zhou, Xianglong Li, Ying Xiao, Jingyao Zhang, Yutao Yang, Li Feng, Y. James Kang

**Affiliations:** ^1^Regenerative Medicine Research Center, West China Hospital, Sichuan University, Chengdu, China; ^2^Department of Neurosurgery, The Affiliated Hospital of Southwest Medical University, Luzhou, China; ^3^Memphis Institute of Regenerative Medicine, University of Tennessee Health Science Center, Memphis, TN, United States

**Keywords:** aging, ischemic stroke, MCAO, hypoxia-inducible factor-1, Cu metabolism MURR domain 3

## Abstract

Elderly patients suffer more brain damage in comparison with young patients from the same ischemic stroke. The present study was undertaken to test the hypothesis that suppressed hypoxia-inducible factor-1 (HIF-1) transcription activity is responsible for defective recovery after ischemic stroke in the elders. Aged and young rats underwent 1-h transient middle cerebral artery occlusion (MCAO) to produce cerebral ischemic injury. The initial cerebral infarct volume in the young gradually declined as time elapsed, but in the aged rats remained the same. The defective recovery in the aged was associated with depressed angiogenesis and retarded neurorestoration. There was no difference in HIF-1α accumulation in the brain between the two age groups, but the expression of HIF-1 regulated genes involved in cerebral recovery was suppressed in the aged. In confirmation, inhibition of HIF-1 transactivation of gene expression in the young suppressed cerebral recovery from MCAO as the same as that observed in the aged rats. Furthermore, a copper metabolism MURR domain 1 (COMMD1) was significantly elevated after MCAO only in the brain of aged rats, and suppression of COMMD1 by siRNA targeting COMMD1 restored HIF-1 transactivation and improved recovery from MCAO-induced damage in the aged brain. These results demonstrate that impaired HIF-1 transcription activity, due at least partially to overexpression of COMMD1, is associated with the defective cerebral recovery from ischemic stroke in the aged rats.

## Introduction

Ischemic stroke accounts for greater than 80% of all strokes and is a major cause of adult disability. Currently, thrombolytic therapy (time window of 4.5 h) and endovascular thrombectomy (time window of 24 h) are effective in the treatment of acute ischemic stroke (Warner et al., 2019). Notably, mortality in the elderly significantly increases in comparison with other age groups treated with thrombolytic or endovascular thrombectomy (Arora et al., 2016; Alawieh et al., 2019).

The incidence of stroke increases with age, which also is an important factor affecting stroke recovery (Badan et al., 2003; Feigin et al., 2003; Popa-Wagner et al., 2006; Petcu et al., 2008; Joseph et al., 2012; Buga et al., 2013; Ciobanu et al., 2017). Although approximately 33% of stroke patients recover spontaneously (Balestrino, 2012), intrinsic self-healing capability in elderly patients is lower than in the young (Hallstrom et al., 2008; Knoflach et al., 2012; Kang and Zheng, 2013). The ability to resist ischemic injury and to recover neurological function is lessened in aged animals (Liu et al., 2016), even though cerebral blood flow does not decrease in aged animals after ischemia (Manwani et al., 2014). Thus, it is imperative to investigate the factors responsible for the increased susceptibility to ischemic/hypoxic injury in the aged brain.

Mammals have an intricate regulatory system for ischemic response, in which hypoxia-inducible factor-1 (HIF-1) plays a critical role (Wiener et al., 1996; Yu et al., 1998). HIF-1 is a heterodimer composed of a HIF-1α subunit (an O_2_-regulated protein) and a HIF-1β subunit (a constitutively expressed protein). Under normoxic conditions, HIF-1α proteins are degraded by the ubiquitin–proteasome pathway, leading to only 5 min short half-life for the protein. In contrast, under hypoxic conditions, the degradation pathway is inhibited, and HIF-1α enters the nucleus, dimerizes with HIF-1β, and ultimately forms a transcription complex with other coactivators to initiate expression of multiple genes (Ke and Costa, 2006). Interestingly, it has been documented that a copper metabolism MURR domain 1 (COMMD1) promotes the degradation of the HIF-1α or competes with HIF-1β for binding to HIF-1α resulting in decreased DNA binding and impaired transcriptional activation of HIF-1 (Van De Sluis et al., 2007, 2010; Muller et al., 2009; Riera-Romo, 2018).

HIF-1 activation serves as a neuroprotectant in young animals following cerebral ischemic injury (Yan et al., 2011; Reischl et al., 2014). Reduced expression of HIF-1 regulated proteins in plasma may impair cerebrovascular function in healthy older volunteers in comparison with healthy young individuals (Sorond et al., 2015), although the underlying mechanism is unclear.

Here, we investigated a possible mechanism for defective recovery of aged brain from ischemic damage relative to young brain focusing on alterations in the regulation of HIF-1 transcription activity. Specifically, we compared HIF-1 activation in the brain between aged and young rats in response to the same cerebral ischemic insult and defined the contribution of suppressed HIF-1 activation to the severity of cerebral ischemic injury due to a defective self-recovery.

## Materials and Methods

### Experimental Animals

Young (3–4 months) and aged (20–24 months) male Sprague–Dawley rats were used for all experiments. Rats were fed food and water *ad libitum*, and kept in a temperature (22–25°C)- and humidity (60–65%)-controlled environment with alternating 12-h cycles of light and dark. The study was approved by the Sichuan Bioethics Committee with all protocols conducted under the Animal Care and Use guidelines. All experiments were performed under the guideline to minimize animal pain and stress throughout the study.

### Surgical Procedure

Transient middle cerebral artery occlusion (MCAO) for 60 min was used to establish acute ischemic stroke, which was followed by reperfusion. During the surgical procedure, rats were anesthetized with a mixture of 1–2% isoflurane in nitric oxide/oxygen (69%/30%) via a face mask. The right common carotid artery (CCA), right internal carotid artery (ICA), and right external carotid artery (ECA) were exposed through a midline incision of the neck. Next, proximal portions of the right CCA and ECA were ligated with 5–0 surgical sutures. A silicone-coated nylon suture (0.34 mm diameter for young rats; 0.43 mm diameter for aged rats; Shadong Biotechnology Ltd., Co., Beijing, China) was inserted to occlude the origin of the right MCA. Reperfusion was produced by gently withdrawing the suture (approximately 10 mm) after MCAO for 60 min to restore blood supply to the MCA territory. Sham-operated animals experienced the same surgical procedure without application of the suture. The body temperature was maintained at 37 ± 0.3°C during the surgical procedures. Rats were euthanized at 5 h, and on day 3, 7, 14, or 21 after MCAO under deep anesthesia. Brain tissue and blood samples were collected for analyses.

### Determination of Infarct Volume

Brain slices (2.0 mm-thick) were incubated in 2% 2,3,5-triphenyltetrazolium chloride (TTC) (Sigma–Aldrich, St. Louis, MO, United States) dissolved in normal saline for 15 min at 37°C, and then transferred into 4% formaldehyde solution for fixation. The area of the left hemisphere (V_1_, deep red staining of normal brain tissue), right hemisphere (V_2_), and infarction (V_3_, white non-staining of the infarct tissue with distinct border in the right side) of each slice was measured (six brain slides per rat) by an analysis system (Photoshop CC; Adobe Systems, San Jose, CA, United States). The infarct volume was calculated using the following equation: [V_1_ – (V_2_ – V_3_)]/V_1_ × 100% (Swanson et al., 1990; O’donnell et al., 2004; Shimamura et al., 2004).

### Neurological Evaluation

Neurological score (4-point scale method) (Bederson et al., 1986; Supplementary Table 1), modified neurological severity score (mNSS) (Chen et al., 2001; Supplementary Table 2), and corner turn test (Zhang et al., 2002) were used for measuring sensorimotor dysfunction following ischemic stroke.

### Quantitation of Microvascular Density

Fluorescein isothiocyanate-dextran (FITCD, Sigma–Aldrich) was used to label the microcirculation and detect the blood capillary density in the cerebral cortex. To calculate the number, volume, length, and branching of blood vessels, 50 μm-thick penumbra sections were imaged using a Nikon A1 confocal system (Nikon, Tokyo, Japan) to create z-stacks. Images were analyzed using the Bitplane Imaris 7.6.3 3D image analysis software package (Bitplane, Belfast, United Kingdom) to create three-dimensional (3D) reconstructions (angiograms) of the peri-infarct cortex vasculature.

### Immunofluorescence of Rat Tissue

The brain was removed and post-fixed in 4% buffered paraformaldehyde for 24 h, followed by cryoprotection in 20% sucrose prepared in 10 mM phosphate-buffered saline (PBS). Brains were cut into 2 mm slices (coronal sections, from bregma + 0.0 to −2 mm) and embedded in optimal cutting temperature medium. Sections (6 μm-thick) were prepared using a cryostat microtome (Leica CM1950; Leica Biosystems, Nussloch, Germany). Frozen sections were fixed in 4% paraformaldehyde at 4°C for 10 min, and then washed three-times for 3 min each with PBS. Sections were permeabilized with 0.1% Triton-X-100 for 10 min in room temperature before blocking. After incubation with PBS-2% BSA for 2 h at 37°C, sections were incubated with primary antibody (mouse anti-HIF-1α, 1:250; Novus Biologicals, Littleton, CO., United States and mouse anti-COMMD1, 1:100; Santa Cruz Biotechnology, Inc., CA, United States) or a mixture of two primary antibodies (rabbit anti-Ki67, 1:500; Thermo Fisher Scientific, Waltham, MA, United States; and mouse anti-rat endothelial cell antigen (RECA), 1:200; Abcam, Cambridge, United Kingdom) or (rabbit anti-Ki67, 1:500; Thermo Fisher Scientific; and mouse anti-NeuN, 1:500; Abcam) at 4°C overnight. After a brief wash with PBS, sections were then incubated with secondary antibody TRITC-conjugated swine anti-rabbit IgG (1:150; Dako, Glostrup, Denmark), TRITC-conjugated donkey anti-mouse IgG (1:500, Abcam), or FITC-conjugated goat anti-mouse IgG (1:150, Invitrogen, Carlsbad, CA, United States) for 1 h at 37°C. After being washing with PBS, sections were incubated with 1:300 4′,6-diamidino-2-phenylindole (DAPI) stain (Invitrogen) for 5 min at room temperature. Fluorescence signals were detected with a Nikon A1 confocal system (Nikon) at excitation/emission wavelengths of 495/519 nm (green), 578/603 nm (red), and 650/668 nm (blue).

### TdT-Mediated dUTP Nick-End Labeling (TUNEL) Assay

Frozen sections were fixed in 4% paraformaldehyde and permeabilized in 0.1% Triton X-100 (in PBS), followed by reaction with enzyme solution (terminal deoxynucleotidyl transferase) and label solution (nucleotide) subsequently (Roche Diagnostics, Mannheim, Germany). Nuclear morphology and the number of nuclei was assessed by DAPI staining. Images were acquired using a Nikon A1 confocal system.

### Microarray Experiment

The cortex or peri-infarct cortex was harvested and rinsed with cold PBS containing diethyl pyrocarbonate for eliminating RNase. Analysis was performed using an Affymetrix GeneChip^®^ Rat Genome 230 2.0 Array (31,000 probe set). RNA sample preparations and microarray hybridization procedures were performed by CapitalBio Corporation (Beijing, China), according to the procedures outlined by Affymetrix.

### GeneChip Data Processing and Analysis

Bioinformatics analysis from raw *CEL* files was performed using Gene Cloud of Biotechnology Information (Genminix Informatics, Shanghai, China), an online platform for microarray data analysis. GeneChip analysis included a series of quality control, background correction, normalization, and summary by robust multiarray average (RMA) (Baze et al., 2010). The difference analysis process, default parameters of the clustering heatmap, and significant analysis of Gene Ontology (GO) and Kyoto Encyclopedia of Genes and Genomes (KEGG) pathways were performed by Gene Cloud of the Biotechnology Information, as previously described (Yang et al., 2016). The false discovery rate (FDR) was applied to monitor the probability of type one error by multiple hypothesis testing. Specifically, differentially expressed genes were selected according to threshold values of ≥1.2 and ≤−1.2-fold change (*p* < 0.05 and FDR < 0.25). First, differences between MCAO-subjected young and aged rats were determined, and then differential expression between sham-operated young and aged rats was then subtracted. Thus, differentially expressed genes between MCAO-subjected young and aged rats obtained using the above calculation were only due to the ischemia model.

Heat map of HIF-1 signaling pathway, as measured by microarray, were shown all groups with Cluster 3.0. Briefly, the normalization and standardization of microarray intensity values within datasets by Z score transformation and the subsequent use of the transformed data to compare multiple genes. Red indicates upregulation of gene expression and green indicates downregulation of gene expression relative to the median for each experiment.

### Quantitative Real-Time PCR (Q-PCR)

TATA-binding protein (TBP) and β-actin were chosen as endogenous controls. The primer sequences were designed using Primer-BLAST of NCBI (Supplementary Table 3). The source RNA used for amplification was derived from the same samples used for microarray hybridization. cDNA was synthesized using 1 μg RNA and PrimeScript^TM^ RT reagent kit (TaKaRa, Dalian, China) in accordance with the manufacturer’s instructions. Briefly, the extracted RNA was dissolved in RNase/DNase-free water, and the reaction conditions were: 37°C for 15 min, 85°C for 5 s (the 20 μL reaction contained 1 μg total RNA, 1 μL PrimeScript RT Enzyme Mix I, 1 μL RT Primer Mix, 4 μL 5 × PrimeScript Buffer 2, and up to 20 μL RNase/DNase-free water). The synthesized cDNA was diluted 20 times with RNase/DNase-free water and stored at −20°C. Q-PCR was performed using SYBR^®^ *Premix Ex Taq*II kit (TaKaRa) with the BIO-RAD CFX96 Real-Time System (Bio-Rad Laboratories, Hercules, CA, United States). The 20 μL reaction system contained 10 μL SYBR^®^ Premix Ex Taq^TM^II, 1 μL Forward Primer (10 μM), 1 μL Reverse Primer (10 μM), 3 μL RNase/DNase-free water, 5 μL cDNA. Cycling conditions included 30 s of denaturation at 95°C, followed by 35 cycles of PCR amplification at 95°C for 5 s, 60°C for 30 s. A melting curve was used to check the specificity for each amplified fragment. For all reactions, triplicate technical and biological repetitions of each individual were performed. Data were analyzed using the 2^–ΔΔ*Ct*^ method with the Bio-Rad CFX96 real-time PCR system.

### Western Blotting

The total protein was extracted from the peri-infarct cortex and quantified using the Thermo Fisher Scientific Pierce bicinchoninic acid protein assay kit. Equal amounts of protein (20 μg) were separated by SDS–PAGE, and proteins were transferred onto polyvinylidene difluoride membranes (Bio-Rad Laboratories). After blocking (TBST with 5% w/v non-fat dry milk), membranes were incubated a mouse monoclonal antibody against HIF-1α (diluted 1:500; Novus Biologicals), a mouse monoclonal antibody against VEGF (diluted 1:1,000; Santa Cruz Biotechnology), or a mouse monoclonal antibody against COMMD1 (diluted 1:1,000; Santa Cruz Biotechnology) followed by HRP-conjugated secondary antibody (diluted 1:2,000; Bio-Rad Laboratories). Membranes were re-incubated with HRP-conjugated monoclonal mouse anti-β-tubulin or anti-GAPDH (diluted 1:2,000; KangChen Biotech, Shanghai, China) as a loading control. Bands visualized by using DAB reagent (Millipore, United States), images were captured and analyzed using Vilber Fusion (VILBER LOURMAT Fusion FX, France).

### Quantitative Analysis Vascular Endothelial Growth Factor (VEGF) in Plasma

Plasma was obtained from rats by centrifuging peripheral blood for 10 min at 2,000 × *g*. Expression of VEGF in plasma was measured using the Milliplex MAP Rat Cytokine/Chemokine Magnetic Bead Panel kit (Cat. RECYTMAG-65K; EMD Millipore, Darmstadt, Germany).

### Chromatin Immunoprecipitation Assay

Portions of frozen tissue samples (300 mg peri-infarct cortex from four rats were combined for each chromatin immunoprecipitation (ChIP); *n* = 4 per group, performed in triplicate) were finely grinded in liquid nitrogen, fixed in 1% formaldehyde for 10 min, and quenched with glycine (0.125 mol/L) for 5 min. After crushing tissue in a Dounce homogenizer with cold 1 × PBS with 0.5% Triton X-100, chromatin was extracted, followed by shearing on a Branson Digital Sonifier instrument (S450D; Branson Ultrasonics, Danbury, CT, United States) for a total of 120 cycles (30% amplitude, 15 s on/off). A small portion of sonicated chromatin was kept as “input” material. The remaining chromatin was incubated overnight with 5 μg of anti-HIF-1α antibody (NB100-123; Novus Biologicals) or normal mouse IgG2b (MAB004; Novus Biologicals) bound to 50 μL Protein-G magnetic beads (10004D, Dynabeads; Invitrogen), followed by extensive washing and elution. Next, immunoprecipitate and input chromatin samples were reverse crosslinked, followed by DNA purification using phenol/chloroform. Real-time PCR amplification of immunoenriched DNA samples was performed using primers for the *Angpt2* promoter (5′-CAGACGTGGTCCTTCGAGTC-3′ and 5′-TGGGGGCTACACTGACTTCT-3′) and *Vegfa* promoter (5′-GCCAGACTCCACAGTGCATA-3′ and 5′-TGTGTGACACTGAGAACGGG-3′). Potential HIF-1 binding sites in the *Angpt2* and *Vegfa* promoters were predicted by the JASPAR database and ALGGEN-PROMO (Messeguer et al., 2002; Farre et al., 2003). Data were normalized by the percentage input method and plotted relative to the IgG control of each sample.

### Administration of the HIF-1 Activity Inhibitor (Chetomin) in Young Rats

Chetomin (final conc. 7 mg/mL; Selleck Chemicals, Houston, TX, United States) was dissolved in an appropriate solution (2% dimethyl sulfoxide, 40% polyethylene glycol 300, 2% Tween 80, and ddH_2_O). Chetomin solution was administered (1 mg/kg body weight) by intraperitoneal injection to young rats immediately following MCAO and once daily thereafter for 7 days. Sham groups received intraperitoneal injections of vehicle solution.

### Treatment of Aged Rats With COMMD1 siRNA

The combination of three COMMD1 siRNA duplex mixture (5 nmol/COMMD1 siRNA duplex, RiboBio Co., Guangzhou, China) or negative control (5 nmol, RiboBio Co., Guangzhou, China) was dissolved in ten microliters of sterile normal saline, and was administered by intracerebroventricular injection to aged rats, as reported previously (Xue et al., 2016). The combined COMMD1 siRNA duplex mixture effectively suppressed COMMD1 protein levels administrated 1 day before MCAO (Supplementary Figure 1). The sequences of the three COMMD1 siRNA are as follows: 5′- AGCAGA TCTTGAAGAAGCT-3′, 5′-GAGGTGGAAGAGAGTATCA-3′, and 5′-TCTGGAGTTTGATGAAGCT-3′.

### Statistical Analysis

All statistical analyses were performed using SPSS software (SPSS Inc., Chicago, IL, United States). Linear regression of infarction volume (Y) on time after ischemic stroke (X), and microvascular density (Y) on time after ischemic stroke (X), was conducted for the slope-ratio and standard curve methods. Overall survival rates were calculated by the Kaplan–Meier method. Data were presented as mean ± SD. To detect differences between means, statistical analyses were performed using unpaired *t*-tests and 1- or 2-factor repeated measures analysis of variance (ANOVA). A value of *p* < 0.05 was considered statistically significant.

## Results

### Defective Recovery From Ischemic Stroke in Aged Rats

**FIGURE 1:**
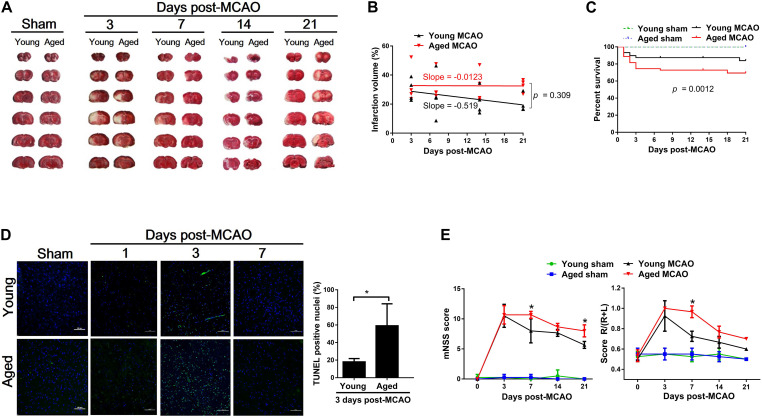
Pathological and functional alterations after MCAO between young and aged rats. **(A)** Coronal rat brain sections (2 mm thick) stained with TTC showing the ischemic core. White reflects the infarct area. **(B)** Changes in brain ischemic infarction area as time elapsed following MCAO between young and aged rats determined by slope-ratio assay. **(C)** Survival rate calculated by Kaplan–Meier curve at 21 days after MCAO. Sham-operated aged (*n* = 20) and young rats (*n* = 20), 100% survival; MCAO young rats (*n* = 90), 85% survival; and MCAO aged rats (*n* = 90), 71.1% survival. **(D)** TUNEL determination in the peri-infarct cortex after MCAO. Data are presented as mean ± SD (n = 4 per group) *p < 0.05 (unpaired *t*-test). **(E)** mNSS and corner turn test at 3, 7, 14, and 21 days after MCAO. Data are presented as mean ± SD (*n* = 3–4 per group). *p < 0.05 (two-way repeated measures ANOVA followed by LSD).

The infarct volume of the brain was measured at 3, 7, 14, or 21 days after transient MCAO in young and aged rats. As shown in Figure 1A, the initial larger infarct volume observed on day 3 in the young decreased from 29.78 ± 7.67% (day 3) to 20.31 ± 6.04% (day 21). However, the cerebral infarct volume in the aged rats remained the same (33.18 ± 13.01% to 32.87 ± 4.26%) throughout the entire period of measurements (Figure 1B). In association with the difference in brain damage, the overall survival rate within 21 days after MCAO was significantly lower in the aged rats (71.1%) than in the young rats (85.6%) (Figure 1C). Furthermore, a significantly larger number of apoptotic cells found in the aged brain than in the young 3 days after MCAO (Figure 1D). Corresponding to these pathological changes, the mNSS test and corner turn test (Figure 1E) showed more retarded movement of the aged rats than the young rats at 7 days after MCAO.

### Depressed Neurorestoration and Angiogenesis in the Aged Rats

**FIGURE 2:**
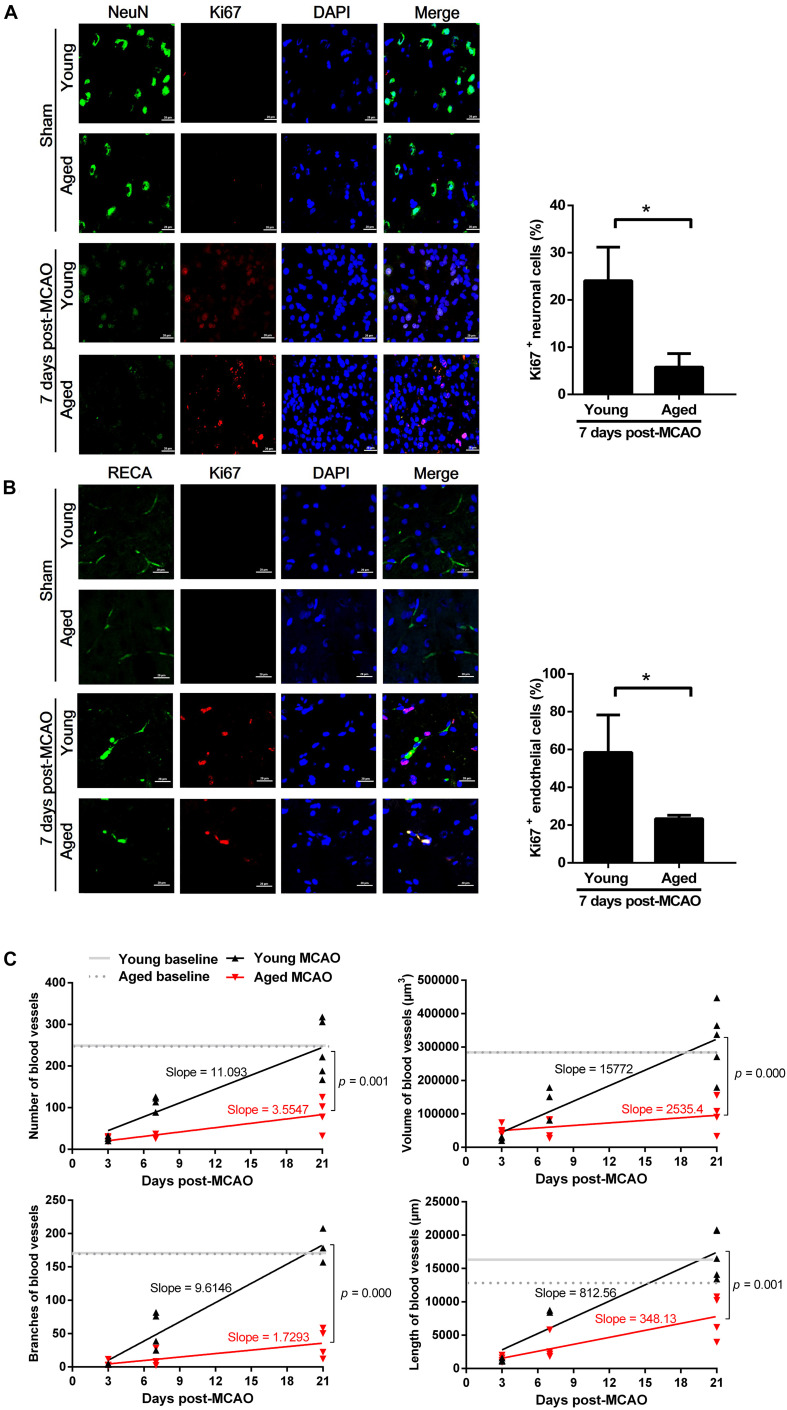
Neurogenesis and angiogenesis in the peri-infarct cortex after MCAO. **(A)** Neurogenesis observed at 7 days after MCAO. Representative confocal image of a peri-infarct cortical section stained with NeuN (neuronal marker, green) and Ki67 (proliferation marker, red). Nuclei were counterstained with DAPI (blue). Data are presented as mean ± SD (*n* = 3 per group). *p < 0.05 (unpaired *t*-test). **(B)** Angiogenesis observed at 7 days after MCAO. Representative confocal image of a peri-infarct cortical section stained with RECA (endothelial cell marker, green) and Ki67 (proliferation marker, red). Nuclei were counterstained with DAPI (blue). Data are presented as mean ± SD (*n* = 3 per group). *p < 0.05 (unpaired *t*-test). **(C)** Microvascular density quantified in the peri-infarct cortex (50 μm-thick sections) before (baseline), and at 3, 7, and 21 days after MCAO. Slope-ratio assay defined the linear regression of microvascular density (Y) against time elapsed (X) after MCAO.

There were much less newly generated neurons in the aged brain (5.79 ± 2.86%) than in the young (24.08 ± 7.11%), as determined by a neuronal nuclei marker (NeuN, green) and a proliferation marker (Ki67, red) on the 7th day after MCAO (Figure 2A). In addition, there was less than 50% of proliferative endothelial cell counts in the aged rat brains (23.35 ± 1.82%) than in the young brains (58.42 ± 19.87%), as defined by an endothelial cell marker (RECA-1, green) and a proliferation marker (Ki67, red), on the 7th day after MCAO (Figure 2B). There was no difference in the cortex vessel density between aged and young rats, as defined by 3D angiograms. In the young rats, the vessel density was significantly decreased 3 days after MCAO, but gradually recovered closing to the baseline level 21 days after MCAO; but in the aged rats this recovery process was significantly suppressed (Figure 2C).

### Alterations in HIF-1 Transcription Activity Between Aged and Young Rats

**FIGURE 3:**
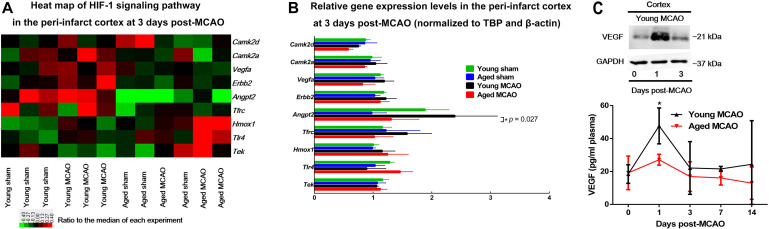
Microarray analysis of HIF-1 related gene expression and relation between VEGF in cortex and in blood. **(A)** Heat map of HIF-1 signaling pathway. A log_2_-transformed expression ratio among four groups. Red indicates up-regulation and green for down-regulation of gene expression relative to the median of all groups. Each group includes three replicates. **(B)** Genes involved in HIF-1 signaling pathway that were validated by quantitative PCR (the RNA used for amplification was derived from the same samples used for microarray). Data are presented as mean ± SD (*n* = 3 per genotype), *p < 0.05 (one-way ANOVA with LSD test), MCAO young rats vs. MCAO aged rats. **(C)** VEGF protein levels in young peri-infarct cortex 0, 1, or 3 days after MCAO (insert); Plasma VEGF concentrations young and aged MCAO rats. Data are shown as mean ± SD from 3 to 6 rats for each group. *p < 0.05 (two-way repeated measures ANOVA followed by LSD), MCAO young rats vs. MCAO aged rats (individual time point).

Gene Ontology (GO) analyses of the brain tissues obtained 3 days after MCAO revealed that there were significant changes in the expression of 5 major categories: (1) genes associated with negative regulation of apoptotic process; (2) organ regeneration; (3) responses to hypoxia; (4) responses to organic cyclic compound; and (5) positive regulation of apoptotic process (Supplementary Tables 3, 4). Among these changes HIF-1 regulated genes were noticeably involved (Figures 3A,B). In particular, the expression of HIF-1 controlled pro-angiogenic genes, *Angpt2* or *Vegfa*, was differentially regulated between aged and young rats.

VEGF protein level was significantly elevated in the young brain, not in the aged, 1 day after MCAO, corresponding to an elevation of blood VEGF levels at the same time in the young rats. In addition, the blood VEGF levels were much lower in the aged than in the young throughout the time period of measurement (Figure 3C).

### Reduced Transcription Activity of HIF-1 in the Brain of Aged Rats

**FIGURE 4:**
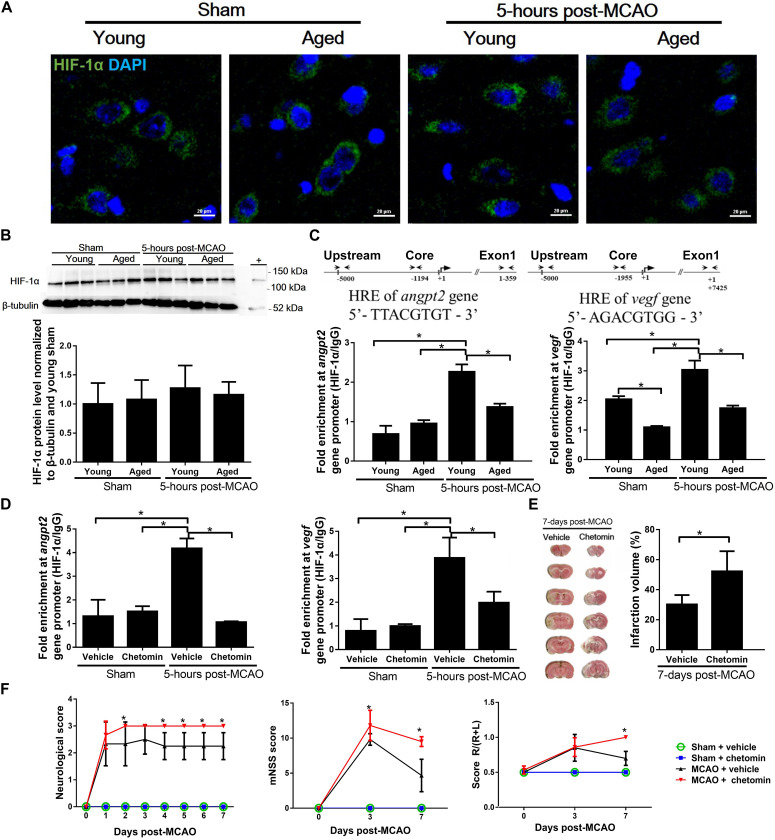
HIF-1α accumulation and transcription activity in peri-infarct cortex after MCAO. **(A)** Immunofluorescence images of peri-infarct cortex. HIF-1α (green) and nuclei counterstained with DAPI (blue) for each group at 5 h after MCAO. **(B)** HIF-1α protein accumulation in peri-infarct cortex and uninjured cortex for each group at 5 h after MCAO. The total protein extracts from hypoxic human umbilical vein endothelial cells were used as positive control (+). Values represent mean ± SD (*n* = 3 per group), all data were analyzed by one-way ANOVA. **(C)** Chromatin immunoprecipitation and quantitative PCR analyses of the peri-infarct cortex for HIF-1 binding to *Angpt2* (–1194 to –1187) and *Vegfa* (–1955 to –1948) promoters 5 h after MCAO in young and aged rats. **(D)** Chromatin immunoprecipitation and quantitative PCR analyses of the peri-infarct cortex for HIF-1 binding to promoters 5 h after MCAO in young rats treated with chetomin or vehicle control. Data were normalized to input. Data are presented as mean ± SD (*n* = 4 per group). *p < 0.05 (one-way ANOVA followed by LSD was performed). **(E)** TTC staining and quantification of infarct volume at 7 days after MCAO in young rats (*n* = 3–6 per group). * *p* < 0.05 (unpaired *t*-tests). **(F)** Neurological score, mNSS, and corner turn test in young rats (*n* = 3–6 per group). *p < 0.05 (two-way repeated measures ANOVA followed by LSD), MCAO + vehicle vs. MCAO + chetomin (individual time point).

To determine changes in HIF-1 transcription activity, both aged and young rat brains were subjected to a 60-min ischemia followed by a 5-h reperfusion before harvesting. There was no difference in HIF-1α protein accumulation in the cortical penumbra between young and aged rats (Figures 4A,B). However, ChIP assay revealed that the binding of HIF-1 to the hypoxic reaction element (HRE) region of the *Angpt2* and *Vegfa* promoters in the aged rats was significantly reduced in comparison to that in the young rats (Figure 4C).

### Diminished Recovery From MCAO by Inhibition of HIF-1 Activity in Young Rats

ChIP analysis showed that the treatment of young rats with chetomin, which interferes with the formation of HIF-1 transcription complex, effectively inhibited the binding of HIF-1 to the HRE region of the *Angpt2* and *Vegfa* promoters (Figure 4D). The MCAO-induced infarct volume in the chetomin-treated young rats was significantly larger than that in the vehicle-treated controls (52.23 ± 13.42% vs. 27.82 ± 2.45%), measured on day 7 after the MCAO (Figure 4E). In addition, there were aggravated neurological deficits in chetomin-treated young rats compared with vehicle-treated rats (Figure 4F). The extent of brain damages from MCAO (infarct volume and neurological score) observed from the chetomin-treated young rats was as the same as that from the aged rats (Figures 1A,E).

### Improved Brain Recovery From MCAO by COMMD1 Deletion in Aged Rats

**FIGURE 5:**
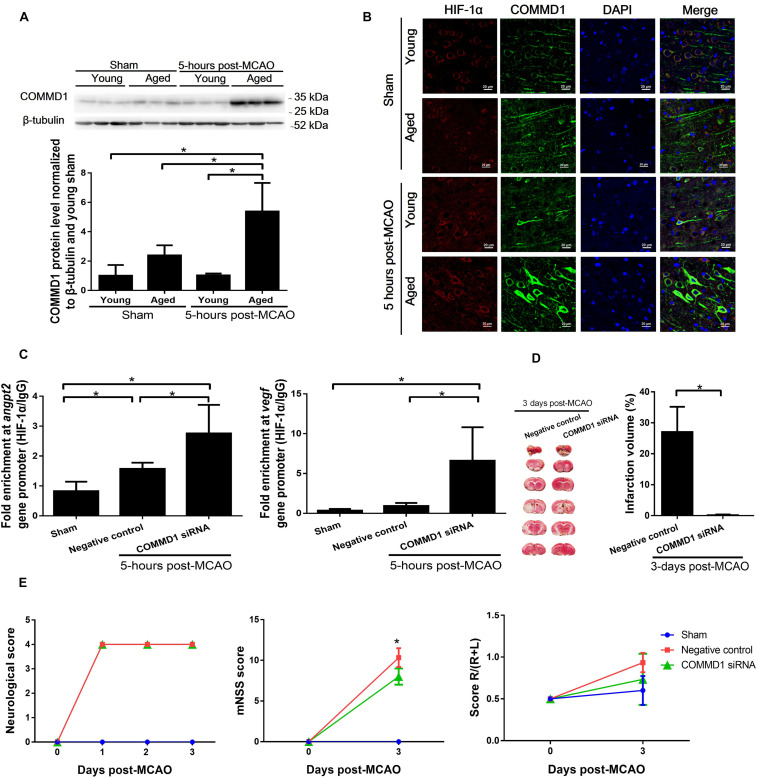
Changes in COMMD1 after MCAO in young and aged rats and effect of siRNA targeting COMMD1 on HIF-1 transactivation of *Angpt2* and *Vegfa* expression and functional recovery after MCAO in aged rats. **(A)** Protein levels of COMMD1 in peri-infarct cortex of MCAO rats at 5 h after MCAO. Values represent mean ± SD (*n* = 3 per group), *p < 0.05 (one-way ANOVA followed by LSD was performed). **(B)** Representative confocal image of a peri-infarct cortical section stained with HIF-1α (red) and COMMD1 (green). Nuclei were counterstained with DAPI (blue). **(C)** Chromatin immunoprecipitation and quantitative PCR analyses of peri-infarct cortex at 5 h after MCAO in aged rats. Data were normalized to input, and presented as mean ± SD (*n* = 4 per group). *p < 0.05 (one-way ANOVA followed by LSD was performed). **(D)** TTC staining and quantification of infarct volume at 3 days MCAO (*n* = 4–5 per group). *p < 0.05 (unpaired *t*-tests). **(E)** Neurological score, mNSS, and corner turn test (*n* = 4–5 per group). *p < 0.05 (two-way repeated measures ANOVA followed by LSD).

COMMD1 protein levels were significantly increased (Figure 5A) in cortical penumbra of aged MCAO rats in comparison to the sham aged and young MCAO rats. The COMMD1 protein was colocalized with HIF-1α in neuronal cells in the ischemic area (Figure 5B, Supplementary Figure 2). The treatment of aged rats with siRNA targeting COMMD1 effectively suppressed COMMD1 protein levels in the brain of these rats (Supplement Figure 1). ChIP analysis showed that COMMD1 deletion in the aged rats increased the binding of HIF-1 to the HRE region of the *Angpt2* and *Vegfa* promoters (Figure 5C). The MCAO-induced infarct volume in the COMMD1 suppressed aged rats was significantly smaller than that in the COMMD1-unchanged controls (2.1 ± 3.9% vs. 25.72 ± 6.81%), measured on day 3 after the MCAO (Figure 5D). The neurological deficits were also diminished in COMMD1 deleted aged rats (Figure 5E).

## Discussion

It has been a long-standing observation, from experimental studies to clinical practice, that aged subjects suffer more brain damage than the young from the same ischemic stroke (Chen and Sun, 2007). It was further defined that defective recovery from the ischemic insult is highly responsible for the ultimate severer structural and functional damage in the aged subjects (Dinapoli et al., 2008). What is responsible for the defective recovery in the elders? The present study specifically, although partially, addressed this question. The depression of HIF-1 regulated angiogenesis is highly associated with the defective recovery from ischemic stroke in the aged.

Both young and aged rats were managed to be subjected to the same ischemic stroke. In our preliminary studies, we found that the surgical suture size affects the extent of MCAO among varying diameters of the MCA in different ages of animals, leading to varying volume sizes of brain damage if the MCAO in different ages of animals were used the same size of sutures. Therefore, we used the suture sizes of 0.43 mm in diameter for the aged and of 0.34 mm for the young to perform the MCAO. This modification led to the same volume of damage in the brain, as measured 3 days after the MCAO, between the young and aged. However, the recovery from the MCAO damage was much better in the young than in the aged, leading to more pathological and functional damages in the aged at the end.

Defective recovery from the ischemic injury in the aged was associated with the suppression of angiogenesis and neurorestoration. That the recovery ability is closely related to angiogenesis and neurogenesis has been demonstrated previously (Zhang et al., 2013; Buga et al., 2014). It was observed in the present study that neororestoration takes place after the MCAO injury. This was indicated by newly generated neurons, as defined by the neurons stained concomitantly by both the neuronal nuclei marker and the proliferation marker. Thus, the self-recovery process of the brain is activated following ischemic injury. The result obtained here clearly shows that in the aged rats, this recovery capacity was significantly diminished. In this recovery process, the most important event is to regrow the damaged vascular system. It was indeed observed that the proliferation of endothelial cells, as defined by the endothelial cell marker in combination with the proliferation marker, was triggered after MCAO, but again, this capacity was also significantly depressed in the aged rats. This depressed proliferation of endothelial cells (a ∼threefold decrease) was accompanied by the decreased capillary density, as defined by the vessel number and volume; and the reduced quality, as defined by the branches and length of the vessels, in the aged in comparison with the young.

What is the underlying mechanism for the depressed angiogenesis in the aged? Angiogenesis in response to ischemia is initially triggered by the activation of transcription factors, which in turn upregulate the expression of pro-angiogenic factors that control the proliferation and migration of endothelial cells to form new blood vessels (Asahara et al., 1997; Carmeliet and Jain, 2000). New neurons are reportedly induced by an ischemia and clustered around newly grown blood vessels (Thored et al., 2007; Masjkur et al., 2012). Therefore, the angiogenesis and neurorestoration is an integrated process that would be proceeded by the involvement of multiple factors. GO analyses of the brain tissues indeed showed significant changes in multiple factors involved in regulation of apoptosis, responses to hypoxia, and organ regeneration. Among these changes, it was particularly noticed that the MCAO-induced expression of pro-angiogenic genes, *Angpt2* or *Vegfa*, was significantly depressed in the aged rats. The critical regulator that controls the expression of these genes is the transcription factor HIF-1.

It was interesting to find that there was no significant difference between aged and young rats in HIF-1α protein accumulation in the brain after a 60-min ischemia followed by 5-h reperfusion. This observation was agreeable with that reported previously, in which both aged and young rats were subjected to 90 min of ischemia followed by 3 days of reperfusion, and there were no significant differences in the HIF-1 signaling pathway between aged and young rats (Buga et al., 2014). In our previous studies (Liu et al., 2018), it was defined that the binding of HIF-1 to the HRE in DNA sequences determines the selectivity of this transcription factor in the regulation of gene expression under different conditions, i.e., not all the HIF-1 controlled genes are simultaneously expressed when the accumulation of HIF-1α protein increases. In this context, the result obtained from the ChIP assay in this study indeed showed that the binding of HIF-1 to the HRE region of the *Angpt2* and *Vegfa* promoters in the aged rats was significantly reduced in comparison to that in the young rats. This difference thus explains why the expression of these two genes were depressed in the aged brain, although there was no significant difference in the accumulation of HIF-1α protein in the brains between the two age groups.

Does inhibition of HIF-1 binding to the HRE region of pro-angiogenic genes affect the recovery of the brain from ischemic stroke? The result obtained from the ChIP analysis of the treatment of young rats with chetomin clearly answered “yes” to this question. Chetomin interferes with the formation of HIF-1 transcription complex, thus effectively inhibiting the binding of HIF-1 to the HRE region of the *Angpt2* and *Vegfa* promoters as presented in the results. The suppression of HIF-1 binding to the HRE did result in defective recovery from MCAO, as indicated by the result that the MCAO-induced infarct volume in the chetomin-treated young rats was significantly larger than that in the vehicle-treated controls. This was further confirmed by the observation that the aggravated neurological deficit in the chetomin-treated young rats was comparable to that observed in the aged rats under the same MCAO treatment.

The ultimate question is why the transcription activity of HIF-1 was suppressed in the aged brain? COMMD1 has been demonstrated to inhibit HIF-1 transcription activity by competing with HIF-1β for binding to HIF-1α in human tumor cells (Muller et al., 2009; Van De Sluis et al., 2010). Interestingly, unlike observations in *Commd1*-deficient embryos (Van De Sluis et al., 2007), observations from tumor cell lines (Muller et al., 2009; Van De Sluis et al., 2010), myocardial infarction mouse model (Li et al., 2020) and our cerebral infarction rats model with reduced expression of COMMD1 did not show increased protein levels of HIF-1α. COMMD1 was indeed significantly increased in aged brain subjected to MCAO, corresponding to suppression of HIF-1 transcription activity in the aged. The cause-and-effect relationship between COMMD1 elevation and suppression of HIF-1 transcription activity in the aged rats was clearly defined by the result using siRNA targeting COMMD1 gene to delete this protein in the brain of the aged. The COMMD1 deletion relieved the suppressive state of HIF-1 binding to the HRE of *Angpt2* and *Vegfa* and greatly improved the recovery of the aged brain from MCAO damage.

There were some troublesome observations related to HIF-1α accumulation in the sham-operated brain and lack of additional HIF-1α accumulation in the MCAO brain in the present study. However, there were previous studies reporting that in the SD rats, there is HIF-1α accumulation in cortex under physiological conditions at various ages (from postnatal day 10–3, 18, 24, and 30 months) (Li et al., 2007; Chen et al., 2012; Wang et al., 2012). We used a model of mild acute ischemic stroke in SD rats; 60-min transient MCAO followed by reperfusion (Liu et al., 2009). This allows middle cerebral artery recanalization instantaneously restoring downstream blood flow in both venues and arterioles (Lin et al., 2008). The results here resemble the results reported in the literature; a 2-h ischemia followed by reperfusion did not change HIF-1α protein accumulation in cortex (Sun et al., 2017).

However, regardless of these seemly unusual observations of HIF-1α accumulation possibly caused by species and experimental procedure differences, the fundamental difference in HIF-1 regulation of angiogenesis between aged and young rats is valid for a partial explanation for defective recovery of aged rats from brain MCAO injury.

There are limitations in the present study: (1) there are multiple factors involved in angiogenesis and neurorestoration. Although we focused on the depressed expression of *Angpt2* and *Vegfa* as possible factors involved in defective recovery from brain injury in the aged rats, other angiogenic factors are also possibly involved and their role will be defined in future studies. (2) The treatment with chetomin to interfere with the binding of HIF-1 to the HRE of *Angpt2* and *Vegfa* would also produce interfering effects on the expression of other transcription factor regulated pro-angiogenic genes. Therefore, the observed suppression of the recovery from MCAO in the young resulting from the chetomin interference would also be ascribed to the inhibition of other pro-angiogenic factors. (3) It remains elusive for the increase in COMMD1 in response to MCAO in the aged rats. Nevertheless, the critical role of transactivation of HIF-1 controlled expression of pro-angiogenic genes in the recovery from MCAO was clearly demonstrated in the present study.

## Conclusion

The present study specifically addressed a possible mechanism for defective recovery from ischemic stroke in aged subjects relative to the young. The results demonstrate that impaired HIF-1 transcription activity, due at least partially to overexpression of COMMD1, is associated with the defective cerebral recovery from ischemic stroke in aged rats.

## Data Availability Statement

The data presented in the study are deposited in the GEO repository, accession number GSE166162.

## Ethics Statement

The animal study was reviewed and approved by Sichuan Bioethics Committee.

## Author Contributions

YG, JZ, and YK conceptualized the study. YG, XL, and YX performed the experiments and analysis for Figures 1, 2. YG and JZ performed the experiments and analysis for Figure 3, performed the experiments for Supplementary Figures 1, 2, and drafted the manuscript and figures. YG, JZ, and YY performed the experiments and analysis for Figures 4, 5. JZ performed GeneChip data processing and analysis for Figures 4, 5. LF edited the manuscript. YK revised and approved the final version of this manuscript. All authors contributed to the article and approved the submitted version.

## Conflict of Interest

The authors declare that the research was conducted in the absence of any commercial or financial relationships that could be construed as a potential conflict of interest.
