# Supplementary material for: The Association of Suppressed Hypoxia-Inducible Factor-1 Transactivation of Angiogenesis With Defective Recovery From Cerebral Ischemic Injury in Aged Rats
Source: Front Aging Neurosci. 2021 Feb 26;13:648115. doi: 10.3389/fnagi.2021.648115 (PMC7953721; doi:10.3389/fnagi.2021.648115)
Supplement: Supplementary file 1 [file Data_Sheet_1.pdf]

Supplementary Figure 1

Knockdown of COMMD1 by siRNA in vivo at 5 hours after MCAO from aged rats.

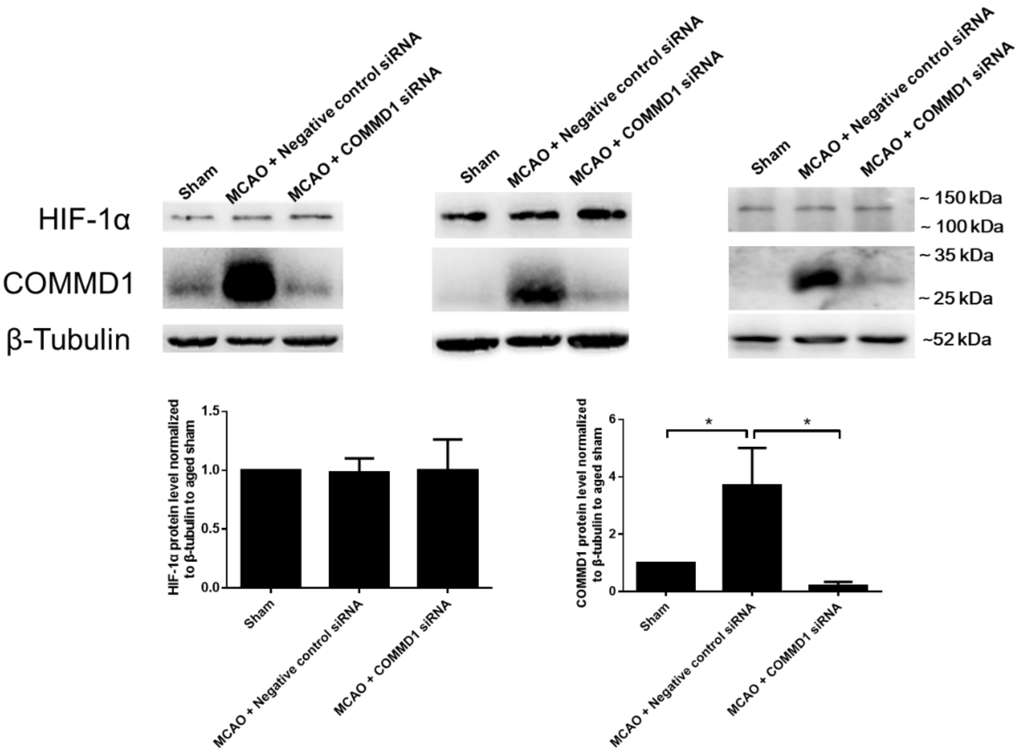

The combination of three COMMD1 siRNA duplex mixture produced knockdown in COMMD1 protein expression at 1 day before MCAO. Data are presented as mean ± SD (n = 3 per group). * $p < 0.05$ (one-way ANOVA followed by LSD).

Supplementary Figure 2

Colocalization of HIF-1α and COMMD1 in neuron nuclei at 5 hours after MCAO from aged rats.

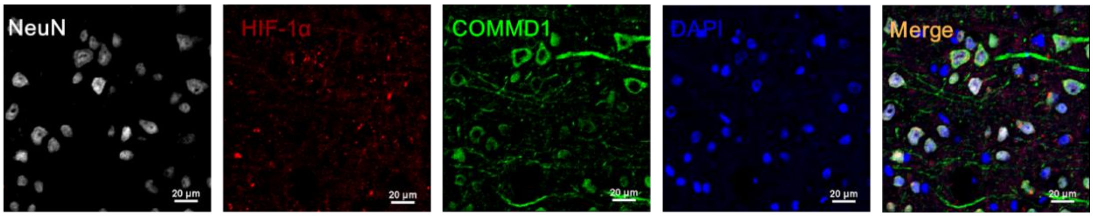

Confocal analysis was performed on brain sections from rat's cortex stained with primary antibody (rabbit anti-NeuN, 1:300; Abcam, goate anti-HIF-1α, 1:300, R&D Systems, and mouse anti-COMMD1, 1:100; Santa Cruz Biotechnology) at 4 °C overnight. After a brief wash with PBS, sections were then incubated with secondary antibody chicken anti-rabbit IgG (H+L) (1:1000, Thermo Fisher) or donkey anti-goat IgG (1:1000, Millipore) or donkey anti-mouse IgG, (1:500, Abcam) for 1 hour at 37 °C. After being washing with PBS, sections were incubated with 1:300 4',6-diamidino-2-phenylindole (DAPI) stain (Invitrogen) for 5 min at room temperature. Fluorescence signals were detected with a Nikon A1 confocal system (Nikon) at excitation/emission wavelengths of 637/700 nm (white), 578/603 nm (red), 495/519 nm (green), and 650/668 nm (blue).

Supplementary Table 1

Neurological score (4-point scale method)

No observable deficit	0
Forelimb flexion	1
Decreased resistance to lateral push without circling	3
Same behavior as grade 2 with circling	4

Supplementary Table 2

Modified Neurological Severity Score Points (mNSS)

Motor tests	
Raising rat by tail	3
Flexion of forelimb	1
Flexion of hindlimb	1
Head moved >10° to vertical axis within 30 s	1
Placing rat on floor (normal = 0; maximum = 3)	3
Normal walk	0
Inability to walk straight	1
Circling toward paretic side	2
Falls down to paretic side	3
Sensory tests	2
Placing test (visual and tactile test)	1
Proprioceptive test (deep sensation, pushing paw against table edge to stimulate limb muscles)	1
Beam balance tests (normal = 0; maximum = 6)	6
Balances with steady posture	0
Grasps side of beam	1
Hugs beam and 1 limb falls down from beam	2
Hugs beam and 2 limbs fall down from beam, or spins on beam (> 60 s)	3
Attempts to balance on beam but falls off (> 40 s)	4
Attempts to balance on beam but falls off (> 20 s)	5
Falls off; no attempt to balance or hang on to beam (< 20 s)	6
Reflex absence and abnormal movements	4
Pinna reflex (head shake when auditory meatus is touched)	1
Corneal reflex (eye blink when cornea is lightly touched with cotton)	1
Startle reflex (motor response to a brief noise from snapping a clipboard paper)	1
Seizures, myoclonus, myodystony	1
Maximum points	18

One point is awarded for inability to perform the tasks or for lack of a tested reflex: 13–18, severe injury; 7–12, moderate injury; 1–6, mild injury.

Supplementary Table 3

Primers used for qRT-PCR

Gene		Primer Sequence
β-actin	sense	5'-TGGCTCCTAGCACCATGAAG-3'
	anti-sense	5'-AAACGCAGCTCAGTAACAGT-3'
TBP	sense	5'-CTGGGCTTCCCAGCTAAGTT-3'
	anti-sense	5'-CTGAACTGCTGGTGGGTCAA-3'
HIF-1α	sense	5'-CGGCGAGAACGAGAAGAAAAATA-3'
	anti-sense	5'-ATGTCAAGATCACCAGCACCTA-3'
Vegfa	sense	5'-AATCCTGGAGCGTTCACTGT-3'
	anti-sense	5'-ACGCGAGTCTGTGTTTTTGC-3'
Angpt2	sense	5'-CATGATGTCATCGCCCGACT-3'
	anti-sense	5'-AAGGTCAGCGTGTAGATGCC-3'
Camk2d	sense	5'-AATGGCATAGTTCACAGGGACC-3'
	anti-sense	5'-GCAAAACCAAACCACGCCT-3'
Erbb2	sense	5'-ATAAGGACACGGAGGGAGAGT-3'
	anti-sense	5'-TGTGTGTCAGGTGAGCAGTAA-3'
Tek	sense	5'-GGGCAAGATGGATAGGGCTC-3'
	anti-sense	5'-GGGCAAGATGGATAGGGCTC-3'
Hmox1	sense	5'-GAAGGCTTTAAGCTGGTGATGG-3'
	anti-sense	5'-GTAGCGGGTATATGCGTGGG-3'
Camk2a	sense	5'-CACCCGATTCACGGAAGAGT-3'
	anti-sense	5'-GGAGTCGGACGATATTGGGG-3'
Tfrc	sense	5'-GTGCTTCAGAGTGCTCCCTTGT-3'
	anti-sense	5'-ACAATAGCCCAGGTAGCCGA-3'
Tlr4	sense	5'-CTCCGCTGGTTGCAGAAAAT-3'
	anti-sense	5'-TTGTCTCAATTTCACACCTGGATAA-3'

Supplementary Table 4

Genes differentially expressed by 60 minutes of ischemia followed 3 days of reperfusion

Probe Set ID	Gene Symbol	Gene Description	Gene Feature
1367517_at	---	---	up
1367541_at	Mettl5	methyltransferase like 5	up
1367553_x_at	Hbb	hemoglobin, beta	up
1367559_at	Ftl	ferritin, light polypeptide	up
1367571_a_at	Igf2	insulin-like growth factor 2	down
1367576_at	Gpx1	glutathione peroxidase 1	up
1367577_at	Hspb1	heat shock protein 1	up
1367592_at	Tnnt2	troponin T type 2 (cardiac)	down
1367627_at	Gatm	glycine amidinotransferase (L-arginine:glycine amidinotransferase)	up
1367646_at	Ctsb	cathepsin B	up
1367648_at	Igfbp2	insulin-like growth factor binding protein 2	down
1367651_at	Ctsd	cathepsin D	up
1367652_at	Igfbp3	insulin-like growth factor binding protein 3	down

Probe Set ID	Gene Symbol	Gene Description	Gene Feature
1367676_at	Hmgb2	high mobility group box 2	up
1367695_at	Qdpr	quinoid dihydropteridine reductase	up
1367700_at	Fmod	fibromodulin	down
1367733_at	Ca2	carbonic anhydrase 2	up
1367748_at	Arf5	ADP-ribosylation factor 5	down
1367776_at	Cdk1	cyclin-dependent kinase 1	up
1367786_at	Psmb8	proteasome (prosome, macropain) subunit, beta type, 8	up
1367813_at	Ppp1r14a	protein phosphatase 1, regulatory (inhibitor) subunit 14A	up
1367846_at	S100a4	S100 calcium-binding protein A4	up
1367847_at	Nupr1	nuclear protein, transcriptional regulator, 1	down
1367850_at	Fcgr2a	Fc fragment of IgG, low affinity IIa, receptor	up
1367851_at	Ptgds	prostaglandin D2 synthase (brain)	down
1367860_a_at	Mmp14	matrix metallopeptidase 14 (membrane-inserted)	down
1367896_at	Car3	carbonic anhydrase 3	up
1367945_at	Atox1	ATX1 antioxidant protein 1 homolog (yeast)	down
1367949_at	Penk	proenkephalin	up
1367961_at	Klk1	kallikrein 1	down
1367973_at	Ccl2	chemokine (C-C motif) ligand 2	up
1367983_at	Fen1	flap structure-specific endonuclease 1	up
1367985_at	Alas2	aminolevulinate, delta-, synthase 2	up
1368000_at	C3	complement component 3	up
1368003_at	Aldh1a2	aldehyde dehydrogenase 1 family, member A2	down
1368006_at	Laptm5	lysosomal protein transmembrane 5	up
1368010_at	Ptpn6	protein tyrosine phosphatase, non-receptor type 6	up
1368064_a_at	Ddc	dopa decarboxylase (aromatic L-amino acid decarboxylase)	up
1368114_at	Fgf13	fibroblast growth factor 13	down
1368124_at	Dusp5	dual specificity phosphatase 5	down
1368127_at	Neu2	sialidase 2	up
1368135_at	Ninj2	ninjurin 2	up
1368136_at	Tmpo	thymopoietin	up
1368146_at	Dusp1	dual specificity phosphatase 1	down
1368161_a_at	Ahsg	alpha-2-HS-glycoprotein	down
1368167_at	Ctse	cathepsin E	up
1368187_at	Gpnmb	glycoprotein (transmembrane) nmb	up
1368223_at	Adamts1	ADAM metallopeptidase with thrombospondin type 1 motif, 1	up
1368244_at	As3mt	arsenic (+3 oxidation state) methyltransferase	up
1368250_at	Tekt1	tektin 1	up
1368255_at	Ntm	neurotrimin	down
1368259_at	Ptgs1	prostaglandin-endoperoxide synthase 1	up
1368270_at	Apobec1	apolipoprotein B mRNA editing enzyme, catalytic polypeptide 1	up
1368280_at	Ctsc	cathepsin C	up
1368293_at	Cpz	carboxypeptidase Z	down
1368300_at	Adora2a	adenosine A2a receptor	up

Probe Set ID	Gene Symbol	Gene Description	Gene Feature
1368302_at	Msx1	msh homeobox 1	down
1368303_at	Per2	period circadian clock 2	down
1368321_at	Egr1	early growth response 1	down
1368329_at	Slc22a6	solute carrier family 22 (organic anion transporter), member 6	down
1368353_at	Gfap	glial fibrillary acidic protein	up
1368359_a_at	Vgf	VGF nerve growth factor inducible	down
1368380_at	Vtn	vitronectin	down
1368384_at	Klk6	kallikrein related-peptidase 6	up
1368395_at	Gpc3	glypican 3	down
1368412_a_at	Ptpro	protein tyrosine phosphatase, receptor type, O	down
1368430_at	Lgmn	legumain	up
1368438_at	Pde10a	phosphodiesterase 10A	up
1368439_at	Sox10	SRY (sex determining region Y)-box 10	up
1368441_at	Msln	mesothelin	down
1368459_at	Gdf10	growth differentiation factor 10	down
1368461_at	Slc22a8	solute carrier family 22 (organic anion transporter), member 8	down
1368462_at	Itpka	inositol-trisphosphate 3-kinase A	down
1368478_at	Drd1	dopamine receptor D1	up
1368490_at	Cd14	CD14 molecule	up
1368495_at	Rln1	relaxin 1	down
1368500_a_at	Rgs9	regulator of G-protein signaling 9	up
1368511_at	Bhlhe41	basic helix-loop-helix family, member e41	up
1368518_at	Cd53	Cd53 molecule	up
1368536_at	Enpp2	ectonucleotide pyrophosphatase/phosphodiesterase 2	up
1368554_at	Pnlip	pancreatic lipase	down
1368559_at	LOC100911216	neuroendocrine convertase 1-like	down
1368582_at	Slc7a3	solute carrier family 7 (cationic amino acid transporter, y+ system), member 3	down
1368599_at	Slc9a2	solute carrier family 9, subfamily A (NHE2, cation proton antiporter 2), member 2	down
1368612_at	Itgb4	integrin, beta 4	down
1368643_at	Spata6	spermatogenesis associated 6	up
1368658_at	Cntf	ciliary neurotrophic factor	up
1368673_at	Ddr2	discoidin domain receptor tyrosine kinase 2	down
1368674_at	Pygl	phosphorylase, glycogen, liver	up
1368677_at	Bdnf	brain-derived neurotrophic factor	down
1368678_at	Bdnf	brain-derived neurotrophic factor	down
1368683_at	Olr1	oxidized low density lipoprotein (lectin-like) receptor 1	up
1368705_at	S1pr5	sphingosine-1-phosphate receptor 5	up
1368708_a_at	Drd2	dopamine receptor D2	up
1368713_at	Mmp10	matrix metallopeptidase 10 (stromelysin 2)	down
1368728_at	P2ry12	purinergic receptor P2Y, G-protein coupled, 12	up
1368732_at	Tap2	transporter 2, ATP-binding cassette, sub-family B (MDR/TAP)	up

Probe Set ID	Gene Symbol	Gene Description	Gene Feature
1368754_at	P2ry6	pyrimidinergic receptor P2Y, G-protein coupled, 6	up
1368770_at	Gcnt1	glucosaminyl (N-acetyl) transferase 1, core 2	up
1368773_at	Cenpi	centromere protein I	up
1368778_at	Slc6a6	solute carrier family 6 (neurotransmitter transporter, taurine), member 6	up
1368834_at	Camk2d	calcium/calmodulin-dependent protein kinase II delta	down
1368839_at	Wfs1	Wolfram syndrome 1 (wolframin)	down
1368858_at	Ugt8	UDP glycosyltransferase 8	up
1368883_at	Nov	nephroblastoma overexpressed	down
1368892_at	Adcyap1	adenylate cyclase activating polypeptide 1	down
1368904_at	Capn10	calpain 10	down
1368908_at	Anxa4	annexin A4	up
1368912_at	Trh	thyrotropin releasing hormone	up
1368914_at	Runx1	runt-related transcription factor 1	up
1368935_at	Camk2a	calcium/calmodulin-dependent protein kinase II alpha	down
1368945_at	Bmp2	bone morphogenetic protein 2	down
1368956_at	Pcdh8	protocadherin 8	down
1368970_at	Cdh23	cadherin-related 23	up
1368971_a_at	Synj2	synaptojanin 2	down
1369001_at	Chrna3	cholinergic receptor, nicotinic, alpha 3 (neuronal)	down
1369018_at	Foxm1	forkhead box M1	up
1369046_at	Syt6	synaptotagmin VI	up
1369054_at	Rph3a	rabphilin 3A	down
1369090_at	Prkg2	protein kinase, cGMP-dependent, type II	down
1369105_a_at	Pkib	protein kinase (cAMP-dependent, catalytic) inhibitor beta	up
1369136_at	Cyp2a3	cytochrome P450, family 2, subfamily a, polypeptide 3	up
1369186_at	Casp1	caspase 1	up
1369200_at	Nt5e	5' nucleotidase, ecto	up
1369221_at	Ppp2r2a	protein phosphatase 2, regulatory subunit B, alpha	down
1369293_at	Rtn4r	reticulon 4 receptor	down
1369334_at	Kcnma1	potassium large conductance calcium-activated channel, subfamily M, alpha member 1	down
1369387_at	Vav1	vav 1 guanine nucleotide exchange factor	up
1369471_at	Fnbp1	formin binding protein 1	up
1369484_at	Wisp2	WNT1 inducible signaling pathway protein 2	down
1369501_at	Zfp260	zinc finger protein 260	down
1369509_a_at	A1bg	alpha-1-B glycoprotein	down
1369530_at	Isl2	ISL LIM homeobox 2	down
1369562_at	Hpcal1	hippocalcin-like 1	down
1369602_at	Fgf17	fibroblast growth factor 17	down
1369660_at	Defb1	defensin beta 1	down
1369665_a_at	Il18	interleukin 18	up
1369691_at	Scn3a	sodium channel, voltage-gated, type III, alpha	down
1369704_at	Slc6a20	solute carrier family 6 (proline IMINO transporter), member 20	down

Probe Set ID	Gene Symbol	Gene Description	Gene Feature
1369705_at	Slc6a20	solute carrier family 6 (proline IMINO transporter), member 20	down
1369713_at	Cckbr	cholecystokinin B receptor	down
1369772_at	Slc6a9	solute carrier family 6 (neurotransmitter transporter, glycine), member 9	up
1369773_at	Bmp3	bone morphogenetic protein 3	down
1369777_a_at	Shank2	SH3 and multiple ankyrin repeat domains 2	down
1369781_at	Grm7	glutamate receptor, metabotropic 7	down
1369792_at	Gpr6	G protein-coupled receptor 6	up
1369919_at	Tef	thyrotrophic embryonic factor	down
1369943_at	Tgm2	transglutaminase 2, C polypeptide	up
1369944_at	LOC100911122	MARCKS-related protein-like	down
1369947_at	Ctsk	cathepsin K	down
1369965_at	Mlh1	mutL homolog 1, colon cancer, nonpolyposis type 2 (E. coli)	up
1369973_at	Xdh	xanthine dehydrogenase	up
1369979_at	Skap2	src kinase associated phosphoprotein 2	up
1370005_at	Cyb5b	cytochrome b5 type B (outer mitochondrial membrane)	up
1370026_at	Cryab	crystallin, alpha B	up
1370028_at	Ace	angiotensin I converting enzyme (peptidyl-dipeptidase A) 1	up
1370047_at	Enpp1	ectonucleotide pyrophosphatase/phosphodiesterase 1	down
1370056_at	LOC100911104	lymphocyte antigen 6B-like	up
1370057_at	Csrp1	cysteine and glycine-rich protein 1	up
1370072_at	Mme	membrane metallo-endopeptidase	up
1370078_at	Lin7b	lin-7 homolog b (C. elegans)	down
1370080_at	Hmox1	heme oxygenase (decycling) 1	up
1370092_at	Mas1	MAS1 oncogene	down
1370118_at	Ccl17	chemokine (C-C motif) ligand 17	down
1370149_at	Asgr1	asialoglycoprotein receptor 1	down
1370157_at	Pln	phospholamban	up
1370186_at	Psmb9	proteasome (prosome, macropain) subunit, beta type, 9	up
1370201_at	Calb1	calbindin 1	down
1370206_at	Asic4	acid-sensing (proton-gated) ion channel family member 4	up
1370216_at	Ddr1	discoidin domain receptor tyrosine kinase 1	up
1370217_at	---	---	up
1370228_at	Srprb	signal recognition particle receptor, B subunit	up
1370239_at	Hba1	hemoglobin, alpha 1	up
1370240_x_at	Hba1	hemoglobin, alpha 1	up
1370247_a_at	Pmp22	peripheral myelin protein 22	up
1370248_at	Fxyd6	FXYD domain-containing ion transport regulator 6	down
1370249_at	Tspo	translocator protein	up
1370290_at	Tubb5	tubulin, beta 5 class I	up
1370320_at	Pbld1	phenazine biosynthesis-like protein domain containing 1	up
1370334_at	Plekhb1	pleckstrin homology domain containing, family B (evectins) member 1	up
1370361_at	Cgref1	cell growth regulator with EF hand domain 1	down
1370369_at	Gzmm	granzyme M (lymphocyte met-ase 1)	down

Probe Set ID	Gene Symbol	Gene Description	Gene Feature
1370371_a_at	Ceacam1	carcinoembryonic antigen-related cell adhesion molecule 1 (biliary glycoprotein)	down
1370387_at	Cyp3a9	cytochrome P450, family 3, subfamily a, polypeptide 9	up
1370388_at	Slc9a5	solute carrier family 9, subfamily A (NHE5, cation proton antiporter 5), member 5	down
1370391_at	Crabp2	cellular retinoic acid binding protein 2	down
1370399_at	Cyp4b1	cytochrome P450, family 4, subfamily b, polypeptide 1	down
1370406_a_at	Cd55	Cd55 molecule	down
1370412_at	Tnnt1	troponin T type 1 (skeletal, slow)	up
1370428_x_at	RT1-A2	RT1 class Ia, locus A2	up
1370449_at	P2ry14	purinergic receptor P2Y, G-protein coupled, 14	up
1370459_at	Aard	alanine and arginine rich domain containing protein	down
1370472_a_at	Kcnma1	potassium large conductance calcium-activated channel, subfamily M, alpha member 1	down
1370477_at	Ocm2	oncomodulin 2	down
1370508_a_at	Cacna1g	calcium channel, voltage-dependent, T type, alpha 1G subunit	down
1370516_at	Slc15a3	solute carrier family 15, member 3	up
1370518_a_at	Stxbp1	syntaxin binding protein 1	down
1370520_at	LOC257650	hippyragranin	up
1370562_at	Calcb	calcitonin-related polypeptide, beta	up
1370577_at	Zfp455	zinc finger protein 455	up
1370595_a_at	Kcnip4	Kv channel interacting protein 4	down
1370605_s_at	Lepr	leptin receptor	down
1370607_a_at	Nrg1	neuregulin 1	up
1370613_s_at	Ugt1a1	UDP glucuronosyltransferase 1 family, polypeptide A1	up
1370658_a_at	St18	suppression of tumorigenicity 18	up
1370706_a_at	Cyp2j3	cytochrome P450, family 2, subfamily j, polypeptide 3	up
1370799_at	Slc9a1	solute carrier family 9, subfamily A (NHE1, cation proton antiporter 1), member 1	down
1370802_at	Itgb5	integrin, beta 5	up
1370814_at	Dhrs4	dehydrogenase/reductase (SDR family) member 4	up
1370817_at	Sec11c	SEC11 homolog C (S. cerevisiae)	up
1370842_at	Bckdk	branched chain ketoacid dehydrogenase kinase	up
1370843_at	Gng8	guanine nucleotide binding protein (G protein), gamma 8	up
1370849_at	Hapln2	hyaluronan and proteoglycan link protein 2	up
1370853_at	Camk2n1	calcium/calmodulin-dependent protein kinase II inhibitor 1	down
1370864_at	Col1a1	collagen, type I, alpha 1	down
1370885_at	Ctsz	cathepsin Z	up
1370892_at	C4a	complement component 4A (Rodgers blood group)	up
1370927_at	Col12a1	collagen, type XII, alpha 1	down
1370928_at	Litaf	lipopolysaccharide-induced TNF factor	up
1370940_at	Tjp2	tight junction protein 2	up
1370941_at	Pdgfra	platelet derived growth factor receptor, alpha polypeptide	down

Probe Set ID	Gene Symbol	Gene Description	Gene Feature
1370952_at	Gstm2	glutathione S-transferase mu 2	down
1370956_at	Dcn	decorin	down
1370959_at	Col3a1	collagen, type III, alpha 1	down
1370971_at	Myh1	myosin, heavy chain 1, skeletal muscle, adult	down
1370973_at	Scn7a	sodium channel, voltage-gated, type VII, alpha	up
1370987_at	Spn	sialophorin	up
1370988_at	---	---	up
1370991_at	Cml3	camello-like 3	up
1371004_at	Sort1	sortilin 1	up
1371019_at	Trib1	tribbles homolog 1 (Drosophila)	down
1371046_at	Sptbn1	spectrin, beta, non-erythrocytic 1	up
1371074_a_at	Mcm6	minichromosome maintenance complex component 6	up
1371079_at	Fcgr2b	Fc fragment of IgG, low affinity IIb, receptor (CD32)	up
1371091_at	Irs2	insulin receptor substrate 2	down
1371102_x_at	LOC100134871	beta globin minor gene	up
1371117_at	Adam32	ADAM metallopeptidase domain 32	down
1371125_at	Kif2a	kinesin heavy chain member 2A	down
1371159_a_at	Cckbr	cholecystokinin B receptor	down
1371194_at	Tnfaip6	tumor necrosis factor alpha induced protein 6	down
1371235_at	Grm6	glutamate receptor, metabotropic 6	down
1371262_at	IgG-2a	gamma-2a immunoglobulin heavy chain	down
1371303_at	Tdh	L-threonine dehydrogenase	up
1371440_at	B2m	beta-2 microglobulin	up
1371518_at	Nid1	nidogen 1	down
1371530_at	Krt8	keratin 8	down
1371545_at	Pecam1	platelet/endothelial cell adhesion molecule 1	up
1371575_at	Msn	moesin	up
1371595_at	LOC100912446	uncharacterized LOC100912446	up
1371616_at	---	---	up
1371668_at	---	---	up
1371671_at	---	---	up
1371672_at	Cbx7	chromobox homolog 7	up
1371700_at	LOC100911714	microfibril-associated glycoprotein 4-like	down
1371762_at	Rbp4	retinol binding protein 4, plasma	down
1371847_at	---	---	up
1371861_at	Sfrp1	secreted frizzled-related protein 1	down
1371862_at	Rrm1	ribonucleotide reductase M1	up
1371959_at	Hist2h2aa3	histone cluster 2, H2aa3	down
1372000_at	Net1	neuroepithelial cell transforming 1	down
1372027_at	---	---	up
1372072_at	Hdhd2	haloacid dehalogenase-like hydrolase domain containing 2	up
1372088_at	Ppfibp1	PTPRF interacting protein, binding protein 1 (liprin beta 1)	down
1372096_at	Oxsr1	oxidative-stress responsive 1	up

Probe Set ID	Gene Symbol	Gene Description	Gene Feature
1372112_at	Trim5	tripartite motif-containing 5	up
1372133_at	Rras2	related RAS viral (r-ras) oncogene homolog 2	down
1372144_at	Dnajb5	DnaJ (Hsp40) homolog, subfamily B, member 5	down
1372179_at	Hpcal1	hippocalcin-like 1	down
1372181_at	Rpa1	replication protein A1	up
1372195_at	Tnnc2	troponin C type 2 (fast)	up
1372208_at	Ppp1r1b	protein phosphatase 1, regulatory (inhibitor) subunit 1B	up
1372234_at	Lix1l	Lix1 homolog (mouse)-like	up
1372246_at	Ostf1	osteoclast stimulating factor 1	up
1372299_at	Cdkn1c	cyclin-dependent kinase inhibitor 1C	down
1372310_at	Isoc1	isochorismatase domain containing 1	up
1372330_at	LOC652955	goliath	up
1372359_at	Dusp26	dual specificity phosphatase 26 (putative)	down
1372382_at	Prkag2	protein kinase, AMP-activated, gamma 2 non-catalytic subunit	down
1372404_at	Rac2	ras-related C3 botulinum toxin substrate 2 (rho family, small GTP binding protein Rac2)	up
1372406_at	Mcm3	minichromosome maintenance complex component 3	up
1372423_at	Perp	PERP, TP53 apoptosis effector	down
1372444_at	---	---	up
1372449_at	Slc8a1	solute carrier family 8 (sodium/calcium exchanger), member 1	up
1372457_at	Mtus1	microtubule associated tumor suppressor 1	up
1372531_at	Ppfibp2	PTPRF interacting protein, binding protein 2 (liprin beta 2)	up
1372563_at	RGD1308143	similar to D330021B20 protein	up
1372587_at	Emcn	endomucin	up
1372601_at	Atf5	activating transcription factor 5	up
1372603_at	Pacsin3	protein kinase C and casein kinase substrate in neurons 3	up
1372616_at	---	---	up
1372633_at	Spg20	spastic paraplegia 20 (Troyer syndrome)	up
1372639_at	Trim54	tripartite motif-containing 54	down
1372641_at	Alpk3	alpha-kinase 3	up
1372646_at	RGD1305645	similar to RIKEN cDNA 1500015O10	up
1372685_at	Cdkn3	cyclin-dependent kinase inhibitor 3	up
1372744_at	Pkp4	plakophilin 4	up
1372750_at	---	---	down
1372791_at	Wnk1	WNK lysine deficient protein kinase 1	up
1372807_at	LOC100912498	protein FAM131A-like	down
1372809_at	LOC290595	hypothetical gene supported by AF152002	up
1372825_at	Fnbp1	formin binding protein 1	up
1372844_at	Efna1	ephrin A1	up
1372846_at	Cyb561a3	cytochrome b561 family, member A3	up
1372858_at	LOC100911740	uncharacterized LOC100911740	down
1372901_at	---	---	up
1372903_at	Kif18b	kinesin family member 18B	up

Probe Set ID	Gene Symbol	Gene Description	Gene Feature
1372905_at	Vcl	vinculin	up
1372930_at	Sp110	SP110 nuclear body protein	up
1372975_at	Lor	loricrin	up
1373008_x_at	Rtn4r	reticulon 4 receptor	down
1373010_at	Krt12	keratin 12	down
1373026_at	Spc24	SPC24, NDC80 kinetochore complex component, homolog (S. cerevisiae)	up
1373043_at	Sdf2l1	stromal cell-derived factor 2-like 1	up
1373053_at	---	---	up
1373054_at	Slc44a1	solute carrier family 44, member 1	up
1373098_at	Bcas1	breast carcinoma amplified sequence 1	up
1373122_at	Ajuba	ajuba LIM protein	down
1373123_at	Rmi1	RMI1, RecQ mediated genome instability 1, homolog (S. cerevisiae)	up
1373148_at	Cpxm2	carboxypeptidase X (M14 family), member 2	down
1373161_at	Tmem98	transmembrane protein 98	up
1373188_at	Scn4b	sodium channel, voltage-gated, type IV, beta	up
1373250_at	Anln	anillin, actin binding protein	up
1373321_at	Myrf	myelin regulatory factor	up
1373330_at	LOC690323	similar to Myosin-15 (Myosin XV) (Unconventional myosin-15)	up
1373354_at	Rnf165	ring finger protein 165	down
1373357_at	---	---	down
1373386_at	Gjb2	gap junction protein, beta 2	down
1373439_at	---	---	up
1373466_at	Cast	calpastatin	up
1373481_at	R3hdm1	R3H domain containing 1	down
1373485_at	---	---	down
1373501_at	---	---	up
1373523_at	Fcgr3a	Fc fragment of IgG, low affinity IIIa, receptor	up
1373526_at	Atg5	autophagy related 5	down
1373536_at	---	---	up
1373537_at	---	---	up
1373538_at	Usp1	ubiquitin specific peptidase 1	up
1373546_at	Atp11a	ATPase, class VI, type 11A	up
1373557_at	Mcm4	minichromosome maintenance complex component 4	up
1373559_at	---	---	down
1373575_at	Fcer1g	Fc fragment of IgE, high affinity I, receptor for; gamma polypeptide	up
1373592_at	Serpinb9	serpin peptidase inhibitor, clade B (ovalbumin), member 9	up
1373611_at	Il17ra	interleukin 17 receptor A	up
1373624_at	---	---	up
1373634_at	---	---	up
1373642_at	Paqr6	progestin and adipoQ receptor family member VI	up
1373657_at	Slc31a2	solute carrier family 31 (copper transporters), member 2	up
1373658_at	Racgap1	Rac GTPase-activating protein 1	up

Probe Set ID	Gene Symbol	Gene Description	Gene Feature
1373672_at	Serpinb9	serpin peptidase inhibitor, clade B (ovalbumin), member 9	up
1373673_at	---	---	up
1373674_at	Mfap5	microfibrillar associated protein 5	down
1373676_at	---	---	up
1373699_at	---	---	down
1373722_at	Kif20a	kinesin family member 20A	up
1373746_at	Wdr91	WD repeat domain 91	up
1373754_at	---	---	up
1373807_at	Vegfa	vascular endothelial growth factor A	down
1373823_at	Cks2	CDC28 protein kinase regulatory subunit 2	up
1373829_at	Fgfr2	fibroblast growth factor receptor 2	up
1373847_at	Tm4sf1	transmembrane 4 L six family member 1	up
1373856_at	RGD1305627	hypothetical LOC314467	up
1373869_at	Soat1	sterol O-acyltransferase 1	up
1373922_at	---	---	up
1373992_at	MGC108823	similar to interferon-inducible GTPase	up
1374035_at	Rem2	RAS (RAD and GEM) like GTP binding 2	up
1374036_at	Mcm2	minichromosome maintenance complex component 2	up
1374039_at	Car14	carbonic anhydrase 14	up
1374061_at	Cd302	CD302 molecule	up
1374065_at	---	---	down
1374076_at	Rfwd3	ring finger and WD repeat domain 3	up
1374089_at	---	---	down
1374095_at	Galnt6	UDP-N-acetyl-alpha-D-galactosamine:polypeptide N-acetylgalactosaminyltransferase 6 (GalNAc-T6)	up
1374102_at	---	---	up
1374113_at	Baz1a	bromodomain adjacent to zinc finger domain, 1A	up
1374126_at	---	---	up
1374137_at	Elf1	E74-like factor 1	up
1374172_at	Col8a2	collagen, type VIII, alpha 2	down
1374199_at	Dip2a	DIP2 disco-interacting protein 2 homolog A (Drosophila)	up
1374207_at	Angpt2	angiopoietin 2	down
1374284_at	Rassf4	Ras association (RalGDS/AF-6) domain family member 4	up
1374298_at	---	---	up
1374317_at	Pik3r6	phosphoinositide-3-kinase, regulatory subunit 6	up
1374325_at	---	---	down
1374348_at	Otud7b	OTU domain containing 7B	up
1374382_at	---	---	up
1374403_at	Efnb1	ephrin B1	down
1374422_at	RGD1562952	similar to Erbb2 interacting protein isoform 2	up
1374449_at	Cdca3	cell division cycle associated 3	up
1374458_at	---	---	up
1374477_at	Prrx2	paired related homeobox 2	down

Probe Set ID	Gene Symbol	Gene Description	Gene Feature
1374503_at	Pbx3	pre-B-cell leukemia homeobox 3	up
1374506_at	Ddx31	DEAD (Asp-Glu-Ala-Asp) box polypeptide 31	up
1374530_at	---	---	down
1374618_at	---	---	up
1374630_at	Clic3	chloride intracellular channel 3	down
1374672_at	Tnni3k	TNNI3 interacting kinase	down
1374686_at	Slc30a7	solute carrier family 30 (zinc transporter), member 7	up
1374705_at	Col4a5	collagen, type IV, alpha 5	down
1374730_at	Tyrobp	Tyro protein tyrosine kinase binding protein	up
1374734_at	LOC680254	hypothetical protein LOC680254	up
1374758_at	---	---	up
1374759_at	Galnt16	UDP-N-acetyl-alpha-D-galactosamine:polypeptide N-acetylgalactosaminyltransferase 16	down
1374775_at	Mki67	antigen identified by monoclonal antibody Ki-67	up
1374779_at	F13a1	coagulation factor XIII, A1 polypeptide	down
1374784_at	Prtfdc1	phosphoribosyl transferase domain containing 1	up
1374794_at	Kif15	kinesin family member 15	up
1374799_at	Ncapd2	non-SMC condensin I complex, subunit D2	up
1374830_at	Ccdc3	coiled-coil domain containing 3	down
1374850_at	---	---	up
1374855_at	Per1	period circadian clock 1	down
1374872_at	Rasgrp2	RAS guanyl releasing protein 2 (calcium and DAG-regulated)	up
1374889_at	Birc7	baculoviral IAP repeat-containing 7	up
1374902_at	Iqgap3	IQ motif containing GTPase activating protein 3	up
1374934_at	---	---	down
1374942_at	Cpxm2	carboxypeptidase X (M14 family), member 2	down
1374948_at	Tmem106a	transmembrane protein 106A	up
1374958_at	Adpgk	ADP-dependent glucokinase	up
1374959_at	Nqo2	NAD(P)H dehydrogenase, quinone 2	up
1374961_at	Pmf1	polyamine-modulated factor 1	up
1374966_at	Dcx	doublecortin	down
1375006_at	---	---	up
1375010_at	Cd68	Cd68 molecule	up
1375026_at	Calml4	calmodulin-like 4	down
1375028_at	---	---	up
1375030_at	B3galt5	UDP-Gal:betaGlcNAc beta 1,3-galactosyltransferase, polypeptide 5	up
1375043_at	---	---	down
1375099_at	---	---	up
1375205_at	Pcaf	p300/CBP-associated factor	up
1375227_at	---	---	up
1375228_at	Brd2	bromodomain containing 2	down
1375316_at	---	---	down
1375319_at	---	---	up

Probe Set ID	Gene Symbol	Gene Description	Gene Feature
1375348_at	---	---	down
1375377_at	Igsf3	immunoglobulin superfamily, member 3	down
1375420_at	Tp53i11	tumor protein p53 inducible protein 11	down
1375430_at	Mmgt2	membrane magnesium transporter 2	up
1375464_at	Vsig10l	V-set and immunoglobulin domain containing 10 like	up
1375495_at	---	---	down
1375499_at	---	---	up
1375513_at	---	---	up
1375538_at	Vcl	vinculin	up
1375548_at	Efcab14	EF-hand calcium binding domain 14	up
1375600_at	Pigo	phosphatidylinositol glycan anchor biosynthesis, class O	down
1375602_at	Rin2	Ras and Rab interactor 2	up
1375606_at	---	---	down
1375625_at	Paqr8	progestin and adipoQ receptor family member VIII	down
1375646_at	Efcab2	EF-hand calcium binding domain 2	up
1375664_at	---	---	up
1375673_at	---	---	up
1375714_at	RGD1562952	similar to Erbb2 interacting protein isoform 2	up
1375751_at	---	---	down
1375767_at	Plekhg3	pleckstrin homology domain containing, family G (with RhoGef domain) member 3	up
1375771_at	Sidt1	SID1 transmembrane family, member 1	down
1375815_at	---	---	down
1375818_at	Lactb	lactamase, beta	up
1375854_at	Ctnnbip1	catenin, beta-interacting protein 1	down
1375866_at	---	---	down
1375870_a_at	Rbms1	RNA binding motif, single stranded interacting protein 1	up
1375873_at	RGD1561149	similar to mKIAA1522 protein	down
1375917_at	Lilrb4	leukocyte immunoglobulin-like receptor, subfamily B, member 4	up
1375937_a_at	Cenpt	centromere protein T	up
1375943_at	Ccp110	centriolar coiled coil protein 110kDa	up
1375951_at	Thbd	thrombomodulin	down
1376039_at	Aurka	aurora kinase A	up
1376057_at	Pde8a	phosphodiesterase 8A	up
1376059_at	LOC100362458	rCG23949-like	up
1376063_at	Wnt6	wingless-type MMTV integration site family, member 6	down
1376098_a_at	---	---	up
1376102_at	Tmbim1	transmembrane BAX inhibitor motif containing 1	up
1376157_at	---	---	up
1376165_at	Slc24a3	solute carrier family 24 (sodium/potassium/calcium exchanger), member 3	down
1376185_at	Kifc1	kinesin family member C1	up
1376208_at	---	---	down

Probe Set ID	Gene Symbol	Gene Description	Gene Feature
1376231_at	Gins1	GINS complex subunit 1 (Psf1 homolog)	up
1376239_at	Atp6v1c2	ATPase, H+ transporting, lysosomal V1 subunit C2	down
1376362_at	Nptxr	neuronal pentraxin receptor	down
1376413_at	---	---	down
1376457_at	Crispld2	cysteine-rich secretory protein LCCL domain containing 2	down
1376472_at	---	---	up
1376558_at	Shisa7	shisa homolog 7 (Xenopus laevis)	down
1376579_at	Lap3	leucine aminopeptidase 3	up
1376594_at	Vezf1	vascular endothelial zinc finger 1	up
1376611_at	Pola1	polymerase (DNA directed), alpha 1, catalytic subunit	up
1376633_at	---	---	down
1376636_at	Tgfbr1	transforming growth factor, beta receptor 1	up
1376652_at	C1qa	complement component 1, q subcomponent, A chain	up
1376667_at	Cyp26b1	cytochrome P450, family 26, subfamily b, polypeptide 1	down
1376684_at	Dlgap5	discs, large (Drosophila) homolog-associated protein 5	up
1376687_at	Usp1	ubiquitin specific peptidase 1	up
1376689_at	Ikbip	IKBKB interacting protein	up
1376710_at	---	---	up
1376721_at	---	---	down
1376734_at	---	---	down
1376755_at	Rarb	retinoic acid receptor, beta	up
1376758_at	Ing1	inhibitor of growth family, member 1	down
1376777_at	---	---	down
1376781_at	---	---	up
1376784_at	---	---	up
1376787_at	Lingo2	leucine rich repeat and Ig domain containing 2	down
1376790_at	Galc	galactosylceramidase	up
1376791_at	Sowaha	sosondowah ankyrin repeat domain family member A	down
1376816_at	---	---	up
1376868_at	Cobll1	cordon-bleu WH2 repeat protein-like 1	up
1376970_at	Ybey	ybeY metallopeptidase	down
1377023_at	Dusp2	dual specificity phosphatase 2	down
1377092_at	---	---	up
1377102_at	Tmem63a	transmembrane protein 63a	up
1377106_at	---	---	up
1377114_at	---	---	down
1377146_at	Vip	vasoactive intestinal peptide	down
1377148_at	---	---	up
1377151_at	---	---	up
1377155_at	---	---	up
1377158_at	---	---	up
1377173_at	---	---	up
1377176_at	RGD1561328	cysteine and histidine rich 1-like	down

Probe Set ID	Gene Symbol	Gene Description	Gene Feature
1377194_a_at	Ccdc90b	coiled-coil domain containing 90B	up
1377198_at	---	---	up
1377227_at	---	---	down
1377240_at	---	---	down
1377328_at	Mitf	microphthalmia-associated transcription factor	up
1377336_at	Sema3b	sema domain, immunoglobulin domain (Ig), short basic domain, secreted, (semaphorin) 3B	up
1377340_at	Tfpi2	tissue factor pathway inhibitor 2	up
1377342_s_at	Fnbp1	formin binding protein 1	up
1377352_at	---	---	up
1377378_at	Scn5a	sodium channel, voltage-gated, type V, alpha subunit	down
1377379_at	Irf6	interferon regulatory factor 6	up
1377385_at	Arhgap27	Rho GTPase activating protein 27	up
1377403_at	---	---	up
1377404_at	Stc1	stanniocalcin 1	down
1377407_at	Acsm5	acyl-CoA synthetase medium-chain family member 5	up
1377442_at	---	---	down
1377451_at	---	---	down
1377453_at	RGD1309108	similar to hypothetical protein FLJ23554	up
1377518_at	Camk1g	calcium/calmodulin-dependent protein kinase IG	down
1377642_at	Cav2	caveolin 2	up
1377651_at	---	---	up
1377662_at	Pir	pirin (iron-binding nuclear protein)	up
1377669_at	Rab27a	RAB27A, member RAS oncogene family	up
1377671_at	---	---	up
1377697_at	Apbb2	amyloid beta (A4) precursor protein-binding, family B, member 2	up
1377716_at	---	---	down
1377733_at	Ctnnbip1	catenin, beta-interacting protein 1	down
1377743_at	---	---	up
1377744_at	Pstpip2	proline-serine-threonine phosphatase-interacting protein 2	up
1377774_at	---	---	up
1377792_at	---	---	up
1377807_a_at	---	---	down
1377808_at	---	---	down
1377821_at	---	---	up
1377826_at	---	---	up
1377885_at	LOC685203	hypothetical protein LOC685203	up
1377899_at	RGD1304982	similar to RIKEN cDNA 2810025M15	up
1377923_at	Stard8	StAR-related lipid transfer (START) domain containing 8	down
1377943_at	---	---	up
1377952_at	Adpgk	ADP-dependent glucokinase	up
1377954_at	---	---	down
1377964_at	LOC100363708	transmembrane protein 167B-like	up

Probe Set ID	Gene Symbol	Gene Description	Gene Feature
1377974_at	---	---	up
1377982_at	Dtx4	deltex homolog 4 (Drosophila)	down
1377993_at	Gng13	guanine nucleotide binding protein (G protein), gamma 13	down
1378014_at	LOC64038	sertolin	up
1378026_at	---	---	down
1378027_at	Pvrl3	poliovirus receptor-related 3	down
1378028_at	Mad2l1	MAD2 mitotic arrest deficient-like 1 (yeast)	up
1378052_at	---	---	up
1378056_at	Gmnn	geminin	up
1378126_at	Dennd2a	DENN/MADD domain containing 2A	up
1378131_at	Slc9a9	solute carrier family 9, subfamily A (NHE9, cation proton antiporter 9), member 9	up
1378140_at	Arl11	ADP-ribosylation factor-like 11	up
1378143_at	Pdcl	phosducin-like	up
1378165_at	Twist1	twist basic helix-loop-helix transcription factor 1	down
1378173_at	---	---	up
1378199_at	Ncapg2	non-SMC condensin II complex, subunit G2	up
1378248_at	Apbb2	amyloid beta (A4) precursor protein-binding, family B, member 2	up
1378254_at	RGD1561609	similar to TBC1 domain family member 4	up
1378258_at	---	---	down
1378305_at	Tm4sf1	transmembrane 4 L six family member 1	up
1378315_at	---	---	up
1378321_at	Rassf4	Ras association (RalGDS/AF-6) domain family member 4	up
1378361_at	Chd7	chromodomain helicase DNA binding protein 7	up
1378372_at	---	---	up
1378373_at	LOC100911851	intersectin-1-like	up
1378379_at	Cox11	cytochrome c oxidase assembly homolog 11 (yeast)	down
1378440_at	---	---	up
1378445_at	Ercc6l2	excision repair cross-complementing rodent repair deficiency, complementation group 6-like 2	up
1378471_at	Raver2	ribonucleoprotein, PTB-binding 2	down
1378493_at	Fam29a	family with sequence similarity 29, member A	up
1378517_at	---	---	down
1378592_at	Trim59	tripartite motif-containing 59	up
1378606_at	Fam13b	family with sequence similarity 13, member B	up
1378640_at	Uhrf1	ubiquitin-like with PHD and ring finger domains 1	up
1378645_at	---	---	up
1378670_at	---	---	up
1378687_at	---	---	up
1378803_at	Nkx6-2	NK6 homeobox 2	up
1378828_at	---	---	up
1378838_at	---	---	down
1378899_at	Slc35d3	solute carrier family 35, member D3	up

Probe Set ID	Gene Symbol	Gene Description	Gene Feature
1378973_at	Otud7b	OTU domain containing 7B	up
1379070_at	---	---	up
1379124_at	---	---	up
1379126_at	---	---	up
1379177_at	LOC100912318	matrix metalloproteinase-28-like	down
1379222_at	---	---	up
1379261_at	LOC691962	hypothetical protein LOC691962	up
1379304_at	Prkrip1	Prkr interacting protein 1 (IL11 inducible)	down
1379313_at	---	---	up
1379331_at	Tnn	tenascin N	down
1379374_at	Lppr4	lipid phosphate phosphatase-related protein type 4	down
1379384_at	Sp1	Sp1 transcription factor	up
1379390_at	St6galnac2	ST6 (alpha-N-acetyl-neuraminyl-2,3-beta-galactosyl-1,3)-N-acetylgalactosaminide alpha-2,6-sialyltransferase 2	up
1379404_at	---	---	down
1379457_at	Nedd1	neural precursor cell expressed, developmentally down-regulated 1	up
1379464_at	---	---	down
1379472_at	Nadsyn1	NAD synthetase 1	up
1379478_at	Ovol1	ovo-like 1(Drosophila)	up
1379479_at	Kif4a	kinesin family member 4A	up
1379482_at	Tm6sf1	transmembrane 6 superfamily member 1	up
1379497_at	---	---	up
1379549_at	Rnf165	ring finger protein 165	down
1379568_at	Ifit2	interferon-induced protein with tetratricopeptide repeats 2	up
1379571_at	Pkp4	plakophilin 4	up
1379582_a_at	Ccna2	cyclin A2	up
1379659_at	Bmp2k	BMP-2 inducible kinase	up
1379673_at	Uap1l1	UDP-N-acteylglucosamine pyrophosphorylase 1-like 1	up
1379723_at	Crebzf	CREB/ATF bZIP transcription factor	up
1379740_at	---	---	up
1379753_at	Pex5l	peroxisomal biogenesis factor 5-like	up
1379826_at	Ccdc107	coiled-coil domain containing 107	down
1379882_a_at	---	---	down
1379888_at	LOC100912602	uncharacterized LOC100912602	up
1379899_at	---	---	up
1379935_at	Ccl7	chemokine (C-C motif) ligand 7	up
1379957_at	Slfn13	schlafen family member 13	up
1379961_at	---	---	down
1379995_at	---	---	up
1380013_at	Pnpla3	patatin-like phospholipase domain containing 3	down
1380025_at	---	---	down
1380038_at	Vps13c	vacuolar protein sorting 13 homolog C (S. cerevisiae)	up
1380145_at	---	---	up

Probe Set ID	Gene Symbol	Gene Description	Gene Feature
1380196_at	---	---	up
1380250_at	Sned1	sushi, nidogen and EGF-like domains 1	down
1380270_at	Mfi2	antigen p97 (melanoma associated) identified by monoclonal antibodies 133.2 and 96.5	down
1380277_at	Rad51ap1	RAD51 associated protein 1	up
1380297_at	---	---	up
1380312_at	---	---	up
1380321_at	Mtus1	microtubule associated tumor suppressor 1	up
1380329_at	Opalin	oligodendrocytic myelin paranodal and inner loop protein	up
1380353_at	---	---	down
1380365_at	---	---	up
1380377_at	Btk	Bruton agammaglobulinemia tyrosine kinase	up
1380383_at	Arl4d	ADP-ribosylation factor-like 4D	down
1380405_at	42621	septin 8	up
1380465_at	---	---	down
1380542_at	---	---	down
1380637_at	Megf11	multiple EGF-like-domains 11	down
1380692_at	Rbl1	retinoblastoma-like 1 (p107)	up
1380706_at	Fnip2	folliculin interacting protein 2	down
1380775_at	Kif20b	kinesin family member 20B	up
1380822_at	---	---	up
1380833_at	Gpld1	glycosylphosphatidylinositol specific phospholipase D1	up
1380852_at	Ralyl	RALY RNA binding protein-like	down
1380903_at	LOC691170	similar to zinc finger protein 84 (HPF2)	up
1380931_at	---	---	up
1381009_at	---	---	down
1381043_at	---	---	up
1381058_at	---	---	up
1381074_at	Cyp2j3	cytochrome P450, family 2, subfamily j, polypeptide 3	up
1381099_at	---	---	up
1381147_at	---	---	up
1381151_at	---	---	up
1381157_at	---	---	down
1381162_at	42623	septin 10	up
1381182_at	---	---	down
1381203_at	Sh3glb1	SH3-domain GRB2-like endophilin B1	up
1381243_at	---	---	down
1381266_at	---	---	up
1381267_at	---	---	down
1381289_at	Haus8	HAUS augmin-like complex, subunit 8	up
1381290_at	---	---	up
1381329_at	---	---	up
1381333_at	---	---	up

Probe Set ID	Gene Symbol	Gene Description	Gene Feature
1381403_at	Me2	malic enzyme 2, NAD(+)-dependent, mitochondrial	up
1381449_s_at	Tgfa	transforming growth factor alpha	up
1381460_at	Chadl	chondroadherin-like	up
1381461_at	Stard5	StAR-related lipid transfer (START) domain containing 5	up
1381470_at	---	---	down
1381505_at	LOC100911574	uncharacterized LOC100911574	down
1381517_at	---	---	up
1381533_at	Rnd1	Rho family GTPase 1	down
1381714_at	---	---	up
1381715_at	---	---	down
1381719_at	---	---	up
1381754_at	---	---	up
1381756_at	Adh6a	alcohol dehydrogenase 6A (class V)	down
1381790_at	Map4k4	mitogen-activated protein kinase kinase kinase kinase 4	up
1381800_at	---	---	up
1381816_at	Rnls	renalase, FAD-dependent amine oxidase	up
1381817_at	---	---	up
1381846_s_at	---	---	up
1381922_at	Slc5a11	solute carrier family 5 (sodium/glucose cotransporter), member 11	up
1382007_at	Sh3glb1	SH3-domain GRB2-like endophilin B1	up
1382026_at	Arhgap9	Rho GTPase activating protein 9	up
1382027_at	---	---	up
1382045_at	Tbc1d15	TBC1 domain family, member 15	up
1382058_at	Rras2	related RAS viral (r-ras) oncogene homolog 2	down
1382091_at	---	---	down
1382205_at	---	---	down
1382209_at	Ccdc28b	coiled coil domain containing 28B	down
1382211_at	---	---	up
1382212_at	---	---	up
1382221_at	---	---	down
1382222_at	Tmem163	transmembrane protein 163	down
1382263_at	Odf2l	outer dense fiber of sperm tails 2-like	up
1382271_at	Rps6ka5	ribosomal protein S6 kinase, polypeptide 5	up
1382277_at	Ly96	lymphocyte antigen 96	up
1382278_at	LOC100912446	uncharacterized LOC100912446	up
1382283_at	Wipf1	WAS/WASL interacting protein family, member 1	up
1382296_at	---	---	down
1382327_at	Doc2g	double C2-like domains, gamma	up
1382336_at	Jazf1	JAZF zinc finger 1	down
1382346_at	---	---	up
1382371_at	Dram2	DNA-damage regulated autophagy modulator 2	up
1382375_at	---	---	down
1382382_at	Ermn	ermin, ERM-like protein	up

Probe Set ID	Gene Symbol	Gene Description	Gene Feature
1382398_at	Shisa3	shisa homolog 3 (Xenopus laevis)	down
1382404_at	---	---	up
1382412_at	---	---	down
1382414_at	---	---	up
1382419_at	Cenpk	centromere protein K	up
1382493_at	Cenpf	centromere protein F	up
1382568_at	---	---	up
1382571_at	---	---	up
1382587_at	---	---	up
1382592_at	Spdl1	spindle apparatus coiled-coil protein 1	up
1382601_at	---	---	up
1382603_at	---	---	up
1382680_at	---	---	up
1382682_at	---	---	up
1382692_at	Clec7a	C-type lectin domain family 7, member A	up
1382712_at	---	---	down
1382755_at	---	---	up
1382781_at	---	---	down
1382805_at	Myo18a	myosin XVIIIa	up
1382854_at	---	---	down
1382859_at	Rif1	RAP1 interacting factor homolog (yeast)	up
1382868_at	Sema6a	sema domain, transmembrane domain (TM), and cytoplasmic domain, (semaphorin) 6A	down
1382882_x_at	---	---	down
1382907_at	Fam46c	family with sequence similarity 46, member C	up
1382923_at	Syncrip	synaptotagmin binding, cytoplasmic RNA interacting protein	up
1382928_at	Pdzd3	PDZ domain containing 3	down
1382950_at	LOC685067	similar to guanylate binding protein family, member 6	up
1382956_at	LOC361016	similar to RIKEN cDNA 4933406L09	up
1382980_at	---	---	down
1383014_at	---	---	down
1383015_at	Tbc1d10c	TBC1 domain family, member 10C	up
1383117_at	Pxmp4	peroxisomal membrane protein 4	down
1383127_at	---	---	up
1383131_at	Itgb2	integrin, beta 2	up
1383190_at	Elmod2	ELMO/CED-12 domain containing 2	down
1383191_at	Ppp1r1a	protein phosphatase 1, regulatory (inhibitor) subunit 1A	down
1383210_at	---	---	down
1383215_at	Ttc28	tetratricopeptide repeat domain 28	down
1383218_at	LOC100912162	uncharacterized LOC100912162	up
1383249_at	---	---	up
1383259_at	Mpp2	membrane protein, palmitoylated 2 (MAGUK p55 subfamily member 2)	down
1383275_at	---	---	up

Probe Set ID	Gene Symbol	Gene Description	Gene Feature
1383290_at	Spint1	serine peptidase inhibitor, Kunitz type 1	up
1383291_at	C7	complement component 7	down
1383309_at	St3gal6	ST3 beta-galactoside alpha-2,3-sialyltransferase 6	up
1383315_at	Tsku	tsukushi, small leucine rich proteoglycan	down
1383322_at	Rasl11b	RAS-like family 11 member B	down
1383331_at	---	---	up
1383338_at	---	---	up
1383366_at	---	---	up
1383370_at	Fam5b	family with sequence similarity 5, member B	down
1383405_at	Ppp1r26	protein phosphatase 1, regulatory subunit 26	down
1383413_at	Hhatl	hedgehog acyltransferase-like	up
1383456_at	---	---	down
1383471_at	Slc46a3	solute carrier family 46, member 3	up
1383516_at	Fgl2	fibrinogen-like 2	up
1383519_at	---	---	up
1383536_at	Dhfr	dihydrofolate reductase	up
1383539_at	---	---	up
1383567_at	Colec11	collectin sub-family member 11	up
1383578_at	LOC100911267	DNA repair protein RAD51 homolog 1-like	up
1383606_at	Tc2n	tandem C2 domains, nuclear	up
1383610_at	Eya2	eyes absent homolog 2 (Drosophila)	down
1383655_at	Tnip2	TNFAIP3 interacting protein 2	up
1383658_at	Laptm5	lysosomal protein transmembrane 5	up
1383681_at	---	---	down
1383697_at	---	---	up
1383708_at	Itgbl1	integrin, beta-like 1	down
1383719_at	Map7	microtubule-associated protein 7	up
1383741_at	---	---	up
1383747_at	Ect2	epithelial cell transforming sequence 2 oncogene	up
1383767_at	---	---	down
1383794_at	RGD1311080	similar to RIKEN cDNA A930038C07	down
1383814_at	LOC100910374	uncharacterized LOC100910374	down
1383839_at	Spg20	spastic paraplegia 20 (Troyer syndrome)	up
1383851_at	Ccdc96	coiled-coil domain containing 96	down
1383852_at	---	---	down
1383861_at	---	---	down
1383875_at	Upk1b	uroplakin 1B	down
1383916_at	---	---	down
1383935_at	---	---	up
1383939_at	---	---	up
1383948_at	Ttc28	tetratricopeptide repeat domain 28	down
1383962_at	Siva1	SIVA1, apoptosis-inducing factor	up
1383964_at	Ankrd50	ankyrin repeat domain 50	down

Probe Set ID	Gene Symbol	Gene Description	Gene Feature
1383989_at	Sox4	SRY (sex determining region Y)-box 4	down
1384013_at	Chl1	cell adhesion molecule with homology to L1CAM	down
1384042_at	---	---	up
1384049_at	Tfap2b	transcription factor AP-2 beta	down
1384068_at	Ckap2	cytoskeleton associated protein 2	up
1384098_at	Rnf125	ring finger protein 125	up
1384112_at	---	---	up
1384174_at	---	---	up
1384186_at	---	---	up
1384227_at	Ptprk	protein tyrosine phosphatase, receptor type, K	down
1384264_at	Myh14	myosin, heavy chain 14, non-muscle	up
1384276_at	LOC363458	similar to procollagen, type IV, alpha 6	down
1384277_at	Plekhh1	pleckstrin homology domain containing, family H (with MyTH4 domain) member 1	up
1384376_at	Dnajb14	DnaJ (Hsp40) homolog, subfamily B, member 14	up
1384406_at	Ccrl1	chemokine (C-C motif) receptor-like 1	down
1384408_at	---	---	down
1384415_at	Sox7	SRY (sex determining region Y)-box 7	up
1384483_at	---	---	up
1384511_at	Dnajb5	DnaJ (Hsp40) homolog, subfamily B, member 5	down
1384565_at	Ndc1	NDC1 transmembrane nucleoporin	up
1384584_at	---	---	up
1384742_at	Atrx	alpha thalassemia/mental retardation syndrome X-linked	up
1384765_at	Rhbdf2	rhomboid 5 homolog 2 (Drosophila)	up
1384770_at	---	---	down
1384793_at	Dab1	Dab, reelin signal transducer, homolog 1 (Drosophila)	down
1384840_at	---	---	up
1384845_at	Dck	deoxycytidine kinase	up
1384863_at	Cpne7	copine VII	down
1384887_at	LOC100911506	uncharacterized LOC100911506	up
1384907_at	LOC306096	similar to Dachshund homolog 1 (Dach1)	up
1384920_at	---	---	up
1384924_at	Prnd	prion protein 2 (dublet)	down
1384932_at	Cdk14	cyclin-dependent kinase 14	down
1384948_at	---	---	up
1384969_at	---	---	down
1384971_at	Depdc7	DEP domain containing 7	up
1384973_at	Serpinb3	serine protease inhibitor B3	down
1384981_at	Cd6	Cd6 molecule	up
1384988_at	Fbxo5	F-box protein 5	up
1385002_at	---	---	down
1385005_at	Slc22a8	solute carrier family 22 (organic anion transporter), member 8	down
1385027_at	---	---	down

Probe Set ID	Gene Symbol	Gene Description	Gene Feature
1385031_at	---	---	down
1385040_at	Fbxo17	F-box protein 17	down
1385041_at	Sorcs3	sortilin-related VPS10 domain containing receptor 3	down
1385073_at	---	---	down
1385086_at	Bub1	BUB1 mitotic checkpoint serine/threonine kinase	up
1385100_at	---	---	up
1385127_at	Bmp5	bone morphogenetic protein 5	down
1385132_at	---	---	up
1385138_at	---	---	up
1385143_at	RGD1561963	similar to Dedicator of cytokinesis protein 10 (Protein zizimin 3)	up
1385153_at	Zfp68	zinc finger protein 68	up
1385170_at	Fstl5	follistatin-like 5	down
1385191_at	---	---	down
1385213_at	Epsti1	epithelial stromal interaction 1 (breast)	up
1385233_at	---	---	up
1385243_at	---	---	up
1385244_at	Adam4	a disintegrin and metalloprotease domain 4	up
1385252_at	Trim34	tripartite motif-containing 34	up
1385261_s_at	---	---	up
1385269_s_at	Ccdc93	coiled-coil domain containing 93	up
1385274_at	Pcdhb19	protocadherin beta 19	up
1385309_at	Ccl24	chemokine (C-C motif) ligand 24	down
1385321_at	Prmt8	protein arginine methyltransferase 8	down
1385331_at	Ccdc24	coiled-coil domain containing 24	down
1385334_at	---	---	up
1385352_at	Slc16a13	solute carrier family 16, member 13 (monocarboxylic acid transporter 13)	down
1385381_at	---	---	up
1385385_at	---	---	down
1385387_at	Nkx2-2	NK2 homeobox 2	up
1385397_at	Steap4	STEAP family member 4	up
1385411_at	Usp43	ubiquitin specific peptidase 43	down
1385414_at	Cd8a	CD8a molecule	up
1385419_at	Gpr143	G protein-coupled receptor 143	up
1385444_at	---	---	down
1385475_a_at	Stra6	stimulated by retinoic acid 6	up
1385477_at	RGD1563155	similar to RIKEN cDNA 1700054O13	down
1385486_at	Bnc2	basonuclin 2	down
1385504_at	Rffl	ring finger and FYVE-like domain containing E3 ubiquitin protein ligase	up
1385511_at	Sez6l	seizure related 6 homolog (mouse)-like	down
1385521_at	---	---	up
1385547_at	Ermn	ermin, ERM-like protein	up
1385568_at	---	---	down

Probe Set ID	Gene Symbol	Gene Description	Gene Feature
1385569_at	---	---	up
1385583_at	Tyr	tyrosinase	down
1385600_at	---	---	down
1385605_at	Gpr84	G protein-coupled receptor 84	up
1385689_at	---	---	up
1385705_at	Pcbp3	poly(rC) binding protein 3	down
1385739_at	---	---	up
1385740_at	Lrrc27	leucine rich repeat containing 27	down
1385787_at	rnf141	ring finger protein 141	up
1385832_s_at	Sash3	SAM and SH3 domain containing 3	up
1385889_at	---	---	up
1385981_at	Lppr4	lipid phosphate phosphatase-related protein type 4	down
1386002_at	---	---	up
1386009_at	Csf3r	colony stimulating factor 3 receptor (granulocyte)	up
1386037_at	---	---	up
1386038_x_at	---	---	up
1386075_at	Crybg3	beta-gamma crystallin domain containing 3	up
1386102_at	---	---	up
1386269_x_at	Sorl1	sortilin-related receptor, LDLR class A repeats-containing	down
1386304_at	---	---	down
1386309_at	---	---	down
1386348_at	---	---	down
1386420_at	---	---	up
1386540_at	---	---	down
1386566_at	Ppp2r3c	protein phosphatase 2, regulatory subunit B'', gamma	up
1386695_at	---	---	up
1386774_at	Fam5b	family with sequence similarity 5, member B	down
1386879_at	Lgals3	lectin, galactoside-binding, soluble, 3	up
1386881_at	Igfbp3	insulin-like growth factor binding protein 3	down
1386893_at	Grn	granulin	up
1386901_at	Cd36	CD36 molecule (thrombospondin receptor)	down
1386912_at	Pcolce	procollagen C-endopeptidase enhancer	down
1386915_at	Anp32b	acidic (leucine-rich) nuclear phosphoprotein 32 family, member B	up
1386922_at	Ca2	carbonic anhydrase 2	up
1386931_at	Tnni3	troponin I type 3 (cardiac)	up
1386935_at	Nr4a1	nuclear receptor subfamily 4, group A, member 1	down
1386943_at	Pllp	plasmolipin	up
1386948_at	Nes	nestin	up
1386967_at	LOC100911716	rho-related GTP-binding protein RhoQ-like	up
1386968_at	Ppp1r1a	protein phosphatase 1, regulatory (inhibitor) subunit 1A	down
1386969_at	Nrn1	neuritin 1	down
1386974_at	Phldb1	pleckstrin homology-like domain, family B, member 1	up
1386976_at	Cd82	Cd82 molecule	up

Probe Set ID	Gene Symbol	Gene Description	Gene Feature
1386994_at	Btg2	BTG family, member 2	down
1386995_at	Btg2	BTG family, member 2	down
1387018_at	Sorbs2	sorbin and SH3 domain containing 2	down
1387024_at	Dusp6	dual specificity phosphatase 6	down
1387029_at	Cfh	complement factor H	up
1387053_at	Fmo1	flavin containing monooxygenase 1	down
1387068_at	Arc	activity-regulated cytoskeleton-associated protein	down
1387072_at	Wnk1	WNK lysine deficient protein kinase 1	up
1387091_at	Padi2	peptidyl arginine deiminase, type II	up
1387122_at	Plagl1	pleiomorphic adenoma gene-like 1	down
1387125_at	S100a9	S100 calcium binding protein A9	up
1387131_at	Serpini1	serpin peptidase inhibitor, clade I (neuroserpin), member 1	down
1387145_at	Gjb1	gap junction protein, beta 1	up
1387165_at	Maf	v-maf musculoaponeurotic fibrosarcoma oncogene homolog (avian)	up
1387179_at	Adcy8	adenylate cyclase 8 (brain)	down
1387182_at	Gpr37	G protein-coupled receptor 37	up
1387197_at	LOC100910891	osteomodulin-like	down
1387223_at	Aadat	aminoadipate aminotransferase	up
1387227_at	Wipf1	WAS/WASL interacting protein family, member 1	up
1387241_at	Gpr88	G-protein coupled receptor 88	up
1387285_at	Atp2b2	ATPase, Ca++ transporting, plasma membrane 2	down
1387306_a_at	Egr2	early growth response 2	down
1387310_at	Atp2c2	ATPase, Ca++ transporting, type 2C, member 2	down
1387320_a_at	Lrrc7	leucine rich repeat containing 7	down
1387337_at	Apitd1	apoptosis-inducing, TAF9-like domain 1	down
1387339_at	Sepp1	selenoprotein P, plasma, 1	up
1387356_at	Wfs1	Wolfram syndrome 1 (wolframin)	down
1387360_at	Stx1a	syntaxin 1A (brain)	down
1387365_at	Nr1h3	nuclear receptor subfamily 1, group H, member 3	up
1387372_at	Slc6a13	solute carrier family 6 (neurotransmitter transporter, GABA), member 13	down
1387434_at	Slc22a4	solute carrier family 22 (organic cation transporter), member 4	up
1387442_at	Egr4	early growth response 4	down
1387450_at	Tgfa	transforming growth factor alpha	up
1387459_at	Pkib	protein kinase (cAMP-dependent, catalytic) inhibitor beta	up
1387515_at	Nmbr	neuromedin B receptor	down
1387572_at	Psd	pleckstrin and Sec7 domain containing	down
1387590_at	Hrh4	histamine receptor H4	down
1387659_at	Gda	guanine deaminase	down
1387668_at	Ptpn11	protein tyrosine phosphatase, non-receptor type 11	up
1387675_at	Plau	plasminogen activator, urokinase	up
1387788_at	Junb	jun B proto-oncogene	down
1387812_at	Pcsk6	proprotein convertase subtilisin/kexin type 6	up

Probe Set ID	Gene Symbol	Gene Description	Gene Feature
1387813_at	Erbb2	v-erb-b2 erythroblastic leukemia viral oncogene homolog 2, neuro/glioblastoma derived oncogene homolog (avian)	down
1387843_at	Fst	follistatin	down
1387881_at	Kcnv1	potassium channel, subfamily V, member 1	down
1387902_a_at	LOC500183	similar to NGF-binding Ig light chain	down
1387908_at	Rasd1	RAS, dexamethasone-induced 1	down
1387952_a_at	Cd44	Cd44 molecule	up
1387982_at	Tlr4	toll-like receptor 4	up
1388052_a_at	Kcnq3	potassium voltage-gated channel, KQT-like subfamily, member 3	down
1388100_at	Cdk5rap2	CDK5 regulatory subunit associated protein 2	up
1388101_at	Dpysl3	dihydropyrimidinase-like 3	down
1388116_at	Col1a1	collagen, type I, alpha 1	down
1388122_at	Gstp1	glutathione S-transferase pi 1	up
1388232_a_at	Lepr	leptin receptor	down
1388256_at	RT1-A3	RT1 class I, locus A3	down
1388275_at	LOC100912777	T-cell receptor beta chain T17T-22-like	down
1388340_at	Ns5atp9	NS5A (hepatitis C virus) transactivated protein 9	up
1388379_at	Ptpn11	protein tyrosine phosphatase, non-receptor type 11	up
1388393_at	LOC685108	similar to proteolipid protein 2	up
1388460_at	Capg	capping protein (actin filament), gelsolin-like	up
1388462_at	Sbk1	SH3-binding domain kinase 1	down
1388479_at	---	---	down
1388480_at	Gltp	glycolipid transfer protein	up
1388484_at	Ube2c	ubiquitin-conjugating enzyme E2C	up
1388496_at	Flnc	filamin C, gamma	up
1388515_at	---	---	up
1388569_at	Serpinf1	serpin peptidase inhibitor, clade F (alpha-2 antiplasmin, pigment epithelium derived factor), member 1	down
1388596_at	Cotl1	coactosin-like 1 (Dictyostelium)	up
1388606_at	---	---	up
1388608_x_at	Hba1	hemoglobin, alpha 1	up
1388618_at	Nid2	nidogen 2 (osteonidogen)	down
1388619_at	Rdx	radixin	up
1388659_at	Carhsp1	calcium regulated heat stable protein 1	up
1388661_at	Mtfp1	mitochondrial fission process 1	down
1388689_at	Acyp2	acylphosphatase 2, muscle type	up
1388721_at	Hspb8	heat shock protein B8	up
1388739_at	Ubr7	ubiquitin protein ligase E3 component n-recognin 7 (putative)	up
1388740_at	Fermt3	fermitin family member 3	up
1388744_at	Mcm7	minichromosome maintenance complex component 7	up
1388750_at	Tfrc	transferrin receptor	down
1388778_at	---	---	up
1388791_at	Spats2l	spermatogenesis associated, serine-rich 2-like	down

Probe Set ID	Gene Symbol	Gene Description	Gene Feature
1388804_at	Mn1	meningioma 1	down
1388821_at	Trib2	tribbles homolog 2 (Drosophila)	down
1388848_at	RGD1308350	similar to hypothetical protein MGC13251	up
1388879_at	Abi3bp	ABI family, member 3 (NESH) binding protein	down
1388913_at	Ppap2c	phosphatidic acid phosphatase type 2c	up
1388917_at	Myo1d	myosin ID	up
1388930_at	Tmem123	transmembrane protein 123	up
1388944_at	---	---	down
1388991_at	Sestd1	SEC14 and spectrin domains 1	down
1389020_at	LOC686539	similar to immunoglobulin superfamily containing leucine-rich repeat	down
1389100_at	Epm2aip1	EPM2A (laforin) interacting protein 1	down
1389102_at	Dock1	dedicator of cyto-kinesis 1	up
1389108_at	Tbcd	tubulin folding cofactor D	down
1389117_at	Osgep	O-sialoglycoprotein endopeptidase	up
1389121_at	---	---	down
1389155_at	Dos	downstream of Stk11	down
1389176_at	Inpp5f	inositol polyphosphate-5-phosphatase F	up
1389181_at	---	---	up
1389187_at	---	---	up
1389210_at	Lcp1	lymphocyte cytosolic protein 1	up
1389227_at	Rhog	ras homolog family member G	up
1389305_at	Anxa4	annexin A4	up
1389306_at	Matn2	matrilin 2	down
1389324_at	Tie1	tyrosine kinase with immunoglobulin-like and EGF-like domains 1	up
1389351_at	Lrrfip1	leucine rich repeat (in FLII) interacting protein 1	up
1389368_at	Cnksr3	Cnksr family member 3	up
1389371_at	---	---	up
1389375_at	Upf2	UPF2 regulator of nonsense transcripts homolog (yeast)	up
1389378_at	Cdc42ep5	CDC42 effector protein (Rho GTPase binding) 5	up
1389403_at	Bmp7	bone morphogenetic protein 7	down
1389408_at	LOC100359539	ribonucleotide reductase M2 polypeptide	up
1389413_at	Evi2a	ecotropic viral integration site 2A	up
1389423_at	Ddr2	discoidin domain receptor tyrosine kinase 2	down
1389425_at	LOC100360582	5',3'-nucleotidase, cytosolic	up
1389440_at	---	---	up
1389482_at	Krcc1	lysine-rich coiled-coil 1	up
1389486_at	---	---	up
1389489_at	---	---	up
1389490_at	Cd248	CD248 molecule, endosialin	down
1389555_at	Tcf19	transcription factor 19	up
1389566_at	Ccnb2	cyclin B2	up
1389585_at	---	---	down
1389617_at	Elk3	ELK3, member of ETS oncogene family	up

Probe Set ID	Gene Symbol	Gene Description	Gene Feature
1389618_at	---	---	up
1389622_at	Slc25a13	solute carrier family 25 (aspartate/glutamate carrier), member 13	up
1389653_at	Pcdhb9	protocadherin beta 9	up
1389654_at	---	---	up
1389726_at	---	---	down
1389777_at	Ribc1	RIB43A domain with coiled-coils 1	up
1389807_at	RGD1308065	hypothetical LOC287935	down
1389811_at	Rasgef1c	RasGEF domain family, member 1C	down
1389833_at	Mmp24	matrix metallopeptidase 24	down
1389865_at	Toe1	target of EGR1, member 1 (nuclear)	down
1389873_at	Pycard	PYD and CARD domain containing	up
1389966_at	Col6a3	collagen, type VI, alpha 3	down
1389990_at	---	---	up
1389998_at	Nr2f2	nuclear receptor subfamily 2, group F, member 2	down
1390014_at	---	---	down
1390026_at	Bag3	Bcl2-associated athanogene 3	up
1390050_at	Golm1	golgi membrane protein 1	up
1390052_at	LOC100912140	uncharacterized LOC100912140	up
1390109_at	LOC100912996	uncharacterized LOC100912996	up
1390127_at	Dixdc1	DIX domain containing 1	up
1390136_at	---	---	up
1390159_at	Rasgrp3	RAS guanyl releasing protein 3 (calcium and DAG-regulated)	up
1390161_at	Cyyr1	cysteine/tyrosine-rich 1	up
1390174_at	Eml1	echinoderm microtubule associated protein like 1	up
1390227_at	Pcp4l1	Purkinje cell protein 4-like 1	up
1390231_at	---	---	down
1390242_at	---	---	down
1390248_at	---	---	down
1390291_at	---	---	up
1390309_a_at	---	---	up
1390327_at	---	---	up
1390348_at	Folr2	folate receptor 2 (fetal)	up
1390383_at	Plin2	perilipin 2	up
1390404_at	Lama2	laminin, alpha 2	down
1390415_at	Trip13	thyroid hormone receptor interactor 13	up
1390420_at	Cpxm1	carboxypeptidase X (M14 family), member 1	down
1390434_at	Tradd	TNFRSF1A-associated via death domain	up
1390443_at	RGD1563888	similar to DNA segment, Chr 16, ERATO Doi 472, expressed	up
1390459_at	---	---	up
1390467_at	---	---	up
1390480_at	Kif13a	kinesin family member 13A	up
1390495_at	---	---	down
1390518_at	Emid1	EMI domain containing 1	down

Probe Set ID	Gene Symbol	Gene Description	Gene Feature
1390525_a_at	Stra6	stimulated by retinoic acid 6	up
1390527_at	RGD1562114	RGD1562114	up
1390532_at	Slc13a4	solute carrier family 13 (sodium/sulfate symporters), member 4	down
1390545_at	---	---	up
1390562_s_at	---	---	up
1390569_at	Cndp1	carnosine dipeptidase 1 (metallopeptidase M20 family)	up
1390584_at	Rnf165	ring finger protein 165	down
1390653_at	Wdr89	WD repeat domain 89	up
1390659_at	---	---	up
1390687_at	Plek	pleckstrin	up
1390735_at	---	---	up
1390783_at	Abca8a	ATP-binding cassette, subfamily A (ABC1), member 8a	up
1390812_a_at	Rerg	RAS-like, estrogen-regulated, growth-inhibitor	up
1390832_at	Tmcc3	transmembrane and coiled-coil domain family 3	up
1390835_at	Slc47a1	solute carrier family 47, member 1	down
1390837_at	---	---	up
1390839_at	Pqlc3	PQ loop repeat containing 3	up
1390854_at	C2cd2l	C2CD2-like	down
1390866_at	---	---	up
1390899_at	LRRTM1	leucine rich repeat transmembrane neuronal 1	down
1390905_at	---	---	up
1390912_at	Pcp4l1	Purkinje cell protein 4-like 1	up
1390937_at	Ppp1r36	protein phosphatase 1, regulatory subunit 36	up
1390942_at	Peli2	pellino E3 ubiquitin protein ligase family member 2	up
1390947_at	Heatr5a	HEAT repeat containing 5A	up
1390955_at	Panx3	pannexin 3	down
1390958_at	Sertm1	serine-rich and transmembrane domain containing 1	down
1390993_at	Pbld1	phenazine biosynthesis-like protein domain containing 1	up
1391018_at	Myo5c	myosin VC	up
1391047_at	---	---	up
1391067_at	---	---	down
1391071_at	---	---	up
1391123_at	---	---	up
1391147_at	---	---	up
1391148_at	---	---	up
1391169_at	Tmem117	transmembrane protein 117	up
1391228_at	Rsph1	radial spoke head 1 homolog (Chlamydomonas)	down
1391229_at	Camk1g	calcium/calmodulin-dependent protein kinase IG	down
1391258_at	---	---	up
1391302_at	LOC100911946	uncharacterized LOC100911946	down
1391317_at	Ska1	spindle and kinetochore associated complex subunit 1	up
1391323_at	Srprb	signal recognition particle receptor, B subunit	up
1391394_s_at	Pdgfrb	platelet derived growth factor receptor, beta polypeptide	down

Probe Set ID	Gene Symbol	Gene Description	Gene Feature
1391411_at	RGD1560248	similar to formin-like 2 isoform B	up
1391430_at	Cyb5r2	cytochrome b5 reductase 2	up
1391436_at	---	---	up
1391463_at	Ddx58	DEAD (Asp-Glu-Ala-Asp) box polypeptide 58	up
1391481_at	---	---	up
1391511_at	---	---	up
1391524_at	Phip	pleckstrin homology domain interacting protein	down
1391569_at	---	---	up
1391603_at	---	---	up
1391630_at	Tbx18	T-box18	down
1391656_at	Cpm	carboxypeptidase M	up
1391687_at	---	---	up
1391737_at	Ncf4	neutrophil cytosolic factor 4	up
1391740_at	---	---	down
1391829_at	---	---	up
1391925_at	Ccl19	chemokine (C-C motif) ligand 19	down
1391936_a_at	---	---	up
1391938_at	Usp11	ubiquitin specific peptidase 11	down
1391963_at	---	---	up
1391976_at	Six4	SIX homeobox 4	up
1391977_at	---	---	up
1391979_at	---	---	up
1391981_at	---	---	down
1391993_at	Large	like-glycosyltransferase	up
1392007_at	---	---	down
1392037_at	---	---	up
1392040_at	Sass6	spindle assembly 6 homolog (C. elegans)	up
1392073_at	---	---	down
1392074_at	Cped1	cadherin-like and PC-esterase domain containing 1	down
1392105_at	---	---	down
1392108_at	---	---	down
1392169_at	---	---	down
1392171_at	Chi3l1	chitinase 3-like 1 (cartilage glycoprotein-39)	up
1392180_at	Sp1	Sp1 transcription factor	up
1392248_at	---	---	up
1392368_at	---	---	up
1392425_x_at	Luc7l	LUC7-like (S. cerevisiae)	up
1392504_at	LOC500028	hypothetical protein LOC500028	up
1392510_at	Fam180a	family with sequence similarity 180, member A	down
1392525_at	---	---	down
1392545_at	Tfap2b	transcription factor AP-2 beta	down
1392547_at	MGC105649	hypothetical LOC302884	up
1392566_at	---	---	up

Probe Set ID	Gene Symbol	Gene Description	Gene Feature
1392582_at	---	---	down
1392597_at	Aif1l	allograft inflammatory factor 1-like	up
1392599_at	Syap1	synapse associated protein 1	up
1392607_at	Epb4.1	erythrocyte membrane protein band 4.1	up
1392633_at	---	---	up
1392638_at	---	---	up
1392646_at	Ano4	anoctamin 4	up
1392710_at	---	---	up
1392733_at	Mipol1	mirror-image polydactyly 1	up
1392736_at	---	---	up
1392759_at	Trem2	triggering receptor expressed on myeloid cells 2	up
1392778_at	LOC100910594	uncharacterized LOC100910594	down
1392788_at	---	---	down
1392794_at	---	---	up
1392817_at	---	---	down
1392840_at	---	---	up
1392894_at	Fgl2	fibrinogen-like 2	up
1392901_at	Lrrc1	leucine rich repeat containing 1	up
1392916_at	Map7	microtubule-associated protein 7	up
1392954_at	---	---	up
1392965_a_at	LOC100909873	SPARC-related modular calcium-binding protein 2-like	down
1392970_at	Tmem108	transmembrane protein 108	down
1392976_at	Tpm2	tropomyosin 2, beta	up
1392986_at	Strn	striatin, calmodulin binding protein	up
1392990_at	Sox17	SRY (sex determining region Y)-box 17	up
1392993_at	Fam13b	family with sequence similarity 13, member B	up
1393041_at	Smc2	structural maintenance of chromosomes 2	up
1393048_at	Adra2a	adrenoceptor alpha 2A	down
1393067_at	Tek	TEK tyrosine kinase, endothelial	up
1393092_at	---	---	up
1393109_at	---	---	down
1393119_at	---	---	down
1393135_at	---	---	up
1393157_at	---	---	down
1393158_at	Cenpq	centromere protein Q	up
1393167_at	---	---	up
1393168_at	LOC688133	similar to mbt domain containing 1	up
1393196_at	Klhl23	kelch-like family member 23	down
1393229_at	Pmel	premelanosome protein	up
1393250_at	---	---	down
1393252_at	---	---	down
1393259_at	---	---	up
1393265_at	---	---	up

Probe Set ID	Gene Symbol	Gene Description	Gene Feature
1393346_at	Ccdc125	coiled-coil domain containing 125	up
1393347_at	Itgal	integrin, alpha L	up
1393383_at	Arhgdig	Rho GDP dissociation inhibitor (GDI) gamma	down
1393389_at	Nr4a3	nuclear receptor subfamily 4, group A, member 3	down
1393406_at	---	---	up
1393421_at	Pxmp4	peroxisomal membrane protein 4	down
1393553_at	---	---	down
1393561_at	---	---	up
1393563_at	Il1rap	interleukin 1 receptor accessory protein	up
1393575_at	---	---	up
1393581_at	Aspm	asp (abnormal spindle) homolog, microcephaly associated (Drosophila)	up
1393598_at	Ccna1	cyclin A1	up
1393641_at	Blnk	B-cell linker	up
1393674_at	---	---	up
1393677_at	---	---	down
1393682_at	Gpr34	G protein-coupled receptor 34	up
1393684_at	Hells	helicase, lymphoid specific	up
1393708_at	Bhlhe22	basic helix-loop-helix family, member e22	down
1393710_at	Icam5	intercellular adhesion molecule 5, telencephalin	down
1393730_at	Adamts4	ADAM metallopeptidase with thrombospondin type 1 motif, 4	up
1393736_at	---	---	up
1393794_at	---	---	up
1393795_at	Zeb2	zinc finger E-box binding homeobox 2	up
1393796_at	Cep152	centrosomal protein 152	up
1393799_at	Unc5b	unc-5 homolog B (C. elegans)	up
1393804_at	Cspp1	centrosome and spindle pole associated protein 1	up
1393811_at	Ercc6l2	excision repair cross-complementing rodent repair deficiency, complementation group 6-like 2	up
1393816_at	Cenpw	centromere protein W	up
1393818_s_at	Fam71e1	family with sequence similarity 71, member E1	down
1393836_at	Mitf	microphthalmia-associated transcription factor	up
1393842_at	Ccdc77	coiled-coil domain containing 77	down
1393863_at	Cd180	CD180 molecule	up
1393875_at	---	---	up
1393910_at	Fam13a	family with sequence similarity 13, member A	up
1393944_at	Zfp521	zinc finger protein 521	up
1394038_at	Ift81	intraflagellar transport 81 homolog (Chlamydomonas)	up
1394061_at	---	---	up
1394135_at	---	---	down
1394234_x_at	---	---	down
1394245_at	---	---	down
1394252_at	Spock3	sparc/osteonectin, cwcv and kazal-like domains proteoglycan (testican) 3	up
1394258_at	---	---	up

Probe Set ID	Gene Symbol	Gene Description	Gene Feature
1394309_at	Tekt5	tektin 5	down
1394310_at	LOC100909873	SPARC-related modular calcium-binding protein 2-like	down
1394419_at	Arhgap11a	Rho GTPase activating protein 11A	up
1394475_at	---	---	down
1394491_at	---	---	up
1394502_at	---	---	up
1394504_at	---	---	down
1394550_at	LOC691221	similar to CG1998-PA	up
1394600_at	Rgs17	regulator of G-protein signaling 17	down
1394660_at	---	---	down
1394671_at	---	---	up
1394673_at	Cd33	CD33 molecule	up
1394699_at	---	---	up
1394716_at	---	---	up
1394735_at	Catsper2	cation channel, sperm associated 2	up
1394819_at	Sp110	SP110 nuclear body protein	up
1394821_at	---	---	up
1394837_at	---	---	down
1394891_at	---	---	up
1394908_at	---	---	down
1394935_at	Wasf2	WAS protein family, member 2	up
1394943_at	Crebzf	CREB/ATF bZIP transcription factor	up
1394966_at	Lppr5	lipid phosphate phosphatase-related protein type 5	down
1395000_at	---	---	up
1395015_at	F8	coagulation factor VIII, procoagulant component	up
1395049_at	---	---	up
1395073_at	---	---	down
1395088_at	---	---	down
1395101_at	---	---	up
1395106_at	---	---	up
1395109_at	---	---	down
1395176_at	---	---	down
1395184_at	Clec12a	C-type lectin domain family 12, member A	up
1395265_at	Gns	glucosamine (N-acetyl)-6-sulfatase	up
1395359_at	---	---	up
1395365_at	---	---	up
1395372_at	---	---	up
1395374_at	---	---	down
1395404_at	Fn3krp	fructosamine-3-kinase-related protein	up
1395405_at	Arhgap33	Rho GTPase activating protein 33	down
1395431_at	---	---	up
1395442_at	---	---	up
1395447_at	Ermn	ermin, ERM-like protein	up

Probe Set ID	Gene Symbol	Gene Description	Gene Feature
1395455_at	Hnrnph3	heterogeneous nuclear ribonucleoprotein H3 (2H9)	up
1395485_s_at	LOC100909685	unconventional myosin-Ib-like	down
1395542_at	---	---	down
1395601_at	Nudt12	nudix (nucleoside diphosphate linked moiety X)-type motif 12	up
1395651_at	---	---	up
1395655_at	---	---	up
1395661_at	---	---	down
1395718_at	---	---	up
1395744_at	Sppl2a	signal peptide peptidase-like 2A	up
1395774_at	Gpr153	G protein-coupled receptor 153	down
1395781_at	---	---	down
1395788_at	---	---	up
1395804_at	---	---	up
1395924_at	---	---	down
1395931_at	---	---	up
1395944_at	Mis18a	MIS18 kinetochore protein homolog A (S. pombe)	up
1396068_at	---	---	up
1396087_at	Arhgef28	Rho guanine nucleotide exchange factor (GEF) 28	down
1396101_at	Stc1	stanniocalcin 1	down
1396119_at	---	---	up
1396135_at	---	---	up
1396289_at	Mdga1	MAM domain containing glycosylphosphatidylinositol anchor 1	up
1396305_at	---	---	down
1396308_at	LOC688126	hypothetical protein LOC688126	down
1396323_at	LOC681383	similar to Protein C10orf11 homolog	up
1396332_at	---	---	down
1396339_at	---	---	down
1396348_at	---	---	up
1396378_at	---	---	up
1396396_at	---	---	up
1396407_at	Gas8	growth arrest specific 8	down
1396439_at	---	---	up
1396463_at	Pnn	pinin, desmosome associated protein	up
1396491_at	---	---	down
1396567_at	---	---	down
1396782_at	---	---	up
1396787_at	---	---	up
1396800_at	---	---	down
1396833_at	---	---	up
1396884_at	---	---	up
1396899_at	---	---	up
1396950_at	---	---	up
1396968_at	---	---	down

Probe Set ID	Gene Symbol	Gene Description	Gene Feature
1396983_at	---	---	up
1397070_at	---	---	up
1397153_at	---	---	down
1397164_at	LOC100912446	uncharacterized LOC100912446	up
1397263_at	---	---	down
1397317_at	Itgb3	integrin, beta 3	up
1397341_at	Pbk	PDZ binding kinase	up
1397363_at	---	---	down
1397429_at	Creb5	cAMP responsive element binding protein 5	up
1397434_at	---	---	up
1397440_at	---	---	up
1397461_at	Glt8d2	glycosyltransferase 8 domain containing 2	down
1397480_at	---	---	down
1397572_at	---	---	down
1397639_at	---	---	down
1397644_at	Mtap	methylthioadenosine phosphorylase	up
1397700_x_at	---	---	down
1397704_at	---	---	up
1397707_at	---	---	down
1397751_at	---	---	up
1397796_at	---	---	up
1397851_at	---	---	up
1397904_at	LOC100909928	uncharacterized LOC100909928	up
1397924_at	---	---	up
1398246_s_at	Fcgr2a	Fc fragment of IgG, low affinity IIa, receptor	up
1398258_at	Apod	apolipoprotein D	up
1398282_at	Kynu	kynureninase	down
1398299_at	Arhgef11	Rho guanine nucleotide exchange factor (GEF) 11	down
1398304_at	Fzd2	frizzled family receptor 2	down
1398335_at	Ttyh3	tweety homolog 3 (Drosophila)	down
1398411_at	---	---	up
1398460_at	RGD1311723	similar to KIAA1731 protein	up
1398482_at	Bcl3	B-cell CLL/lymphoma 3	up
1398501_at	Lrrc61	leucine rich repeat containing 61	up
1398505_at	Rasip1	Ras interacting protein 1	down
1398525_at	---	---	down
1398560_at	---	---	up
1398582_at	Rps6ka5	ribosomal protein S6 kinase, polypeptide 5	up
1398595_at	Rbm5	RNA binding motif protein 5	up
1398618_s_at	---	---	up
1398620_at	---	---	up
1398695_at	---	---	up
1398716_at	---	---	down

Probe Set ID	Gene Symbol	Gene Description	Gene Feature
1398756_at	LOC300303	similar to Nucleophosmin (NPM) (Nucleolar phosphoprotein B23) (Numatrin) (Nucleolar protein NO38)	up
1398847_at	Nudt4	nudix (nucleoside diphosphate linked moiety X)-type motif 4	down
1398892_at	Npc2	Niemann-Pick disease, type C2	up
1399069_at	---	---	up
1399083_at	Abhd11	abhydrolase domain containing 11	down
1399107_at	Smim8	small integral membrane protein 8	up
1399164_a_at	Smim8	small integral membrane protein 8	up
1399167_a_at	Gab1	GRB2-associated binding protein 1	up

Supplementary Table 5

GO enrichment analysis by 60 minutes of ischemia followed 3 days of reperfusion

Rank	GO Name	p-value	FDR
1	negative regulation of apoptotic process	1.25E-15	3.45E-12
2	organ regeneration	1.02E-11	1.25E-08
3	response to hypoxia	1.36E-11	1.25E-08
4	response to organic cyclic compound	2.66E-11	1.83E-08
5	positive regulation of apoptotic process	5.78E-11	3.18E-08
6	locomotory behavior	2.54E-10	1.01E-07
7	cellular response to lipopolysaccharide	2.58E-10	1.01E-07
8	cell adhesion	3.78E-10	1.30E-07
9	nervous system development	5.18E-10	1.58E-07
10	negative regulation of cell proliferation	5.78E-10	1.59E-07
11	proteolysis	1.09E-09	2.67E-07
12	response to hydrogen peroxide	1.16E-09	2.67E-07
13	response to drug	1.35E-09	2.87E-07
14	response to ethanol	1.59E-09	3.13E-07
15	positive regulation of transcription, DNA-dependent	2.06E-09	3.78E-07
16	wound healing	2.48E-09	4.27E-07
17	angiogenesis	4.84E-09	7.84E-07
18	kidney development	6.54E-09	9.99E-07
19	positive regulation of ERK1 and ERK2 cascade	7.01E-09	9.99E-07
20	Biological process	7.26E-09	9.99E-07
21	positive regulation of transcription from RNA polymerase II promoter	8.45E-09	1.11E-06
22	response to estradiol stimulus	1.26E-08	1.58E-06
23	integrin-mediated signaling pathway	2.40E-08	2.87E-06
24	growth	2.86E-08	3.28E-06
25	cell division	3.19E-08	3.51E-06
26	positive regulation of tumor necrosis factor production	3.81E-08	4.04E-06
27	lung development	4.38E-08	4.47E-06
28	cellular response to organic substance	5.04E-08	4.96E-06
29	DNA replication initiation	5.90E-08	5.60E-06

Rank	GO Name	p-value	FDR
30	cell-matrix adhesion	9.27E-08	8.50E-06
31	negative regulation of transcription from RNA polymerase II promoter	1.12E-07	9.91E-06
32	extracellular matrix organization	1.19E-07	1.02E-05
33	signal transduction	1.73E-07	1.44E-05
34	positive regulation of MAPK cascade	1.83E-07	1.48E-05
35	brain development	2.47E-07	1.94E-05
36	mitotic cell cycle	2.55E-07	1.95E-05
37	response to mechanical stimulus	3.16E-07	2.35E-05
38	DNA unwinding involved in replication	3.67E-07	2.66E-05
39	cell differentiation	4.40E-07	3.11E-05
40	positive regulation of angiogenesis	4.66E-07	3.21E-05
41	regulation of transcription, DNA-dependent	5.40E-07	3.63E-05
42	platelet aggregation	5.97E-07	3.91E-05
43	cell migration	6.32E-07	4.05E-05
44	cell cycle	7.68E-07	4.68E-05
45	aging	7.76E-07	4.68E-05
46	regulation of cell migration	7.82E-07	4.68E-05
47	positive regulation of cell proliferation	1.08E-06	6.34E-05
48	positive regulation of epithelial cell proliferation	1.11E-06	6.39E-05
49	response to toxic substance	1.22E-06	6.86E-05
50	inner ear development	1.30E-06	7.14E-05
51	response to estrogen stimulus	1.53E-06	8.26E-05
52	response to retinoic acid	2.66E-06	0.00014
53	positive regulation of endothelial cell proliferation	2.69E-06	0.00014
54	positive regulation of I-kappaB kinase/NF-kappaB cascade	3.23E-06	0.000165
55	intracellular signal transduction	3.98E-06	0.000199
56	heart development	4.34E-06	0.000213
57	sodium ion transport	5.12E-06	0.000247
58	induction of apoptosis	5.45E-06	0.000259
59	mitosis	5.84E-06	0.000273
60	positive regulation of cell migration	6.00E-06	0.000275
61	negative regulation of cell cycle	6.08E-06	0.000275
62	multicellular organismal development	6.49E-06	0.000288
63	response to progesterone stimulus	7.49E-06	0.000325
64	in utero embryonic development	7.56E-06	0.000325
65	osteoblast differentiation	7.72E-06	0.000327
66	response to glucose stimulus	9.43E-06	0.000393
67	small GTPase mediated signal transduction	9.70E-06	0.000399
68	ossification	1.05E-05	0.000426
69	apoptotic process	1.09E-05	0.000435
70	odontogenesis of dentin-containing tooth	1.16E-05	0.000457
71	endoderm formation	1.18E-05	0.000459
72	cell proliferation	1.33E-05	0.00051

Rank	GO Name	p-value	FDR
73	activation of protein kinase activity	1.37E-05	0.000517
74	negative regulation of transcription, DNA-dependent	1.43E-05	0.000532
75	protein dephosphorylation	1.64E-05	0.000585
76	cellular response to insulin stimulus	1.64E-05	0.000585
77	cellular potassium ion homeostasis	1.68E-05	0.000585
78	positive regulation of cell proliferation by VEGF-activated platelet derived growth factor receptor signaling pathway	1.68E-05	0.000585
79	positive regulation of p38MAPK cascade	1.68E-05	0.000585
80	positive regulation of Wnt receptor signaling pathway	1.77E-05	0.000603
81	vasodilation	1.77E-05	0.000603
82	response to peptide hormone stimulus	2.19E-05	0.000734
83	positive regulation of smooth muscle cell migration	2.27E-05	0.000752
84	sodium ion transmembrane transport	2.46E-05	0.000796
85	response to axon injury	2.46E-05	0.000796
86	cell development	2.86E-05	0.000906
87	embryonic cranial skeleton morphogenesis	2.86E-05	0.000906
88	negative regulation of kinase activity	3.95E-05	0.001236
89	innate immune response	4.07E-05	0.00126
90	peptidyl-tyrosine phosphorylation	4.44E-05	0.001357
91	spermatogenesis	4.82E-05	0.001446
92	cellular response to growth factor stimulus	4.83E-05	0.001446
93	response to nicotine	5.60E-05	0.001659
94	positive regulation of gene expression	5.81E-05	0.001702
95	phosphatidylinositol metabolic process	6.32E-05	0.001812
96	microtubule-based movement	6.46E-05	0.001812
97	memory	6.46E-05	0.001812
98	positive regulation of MAP kinase activity	6.52E-05	0.001812
99	mitral valve morphogenesis	6.61E-05	0.001812
100	positive regulation of adenylate cyclase activity involved in G-protein coupled receptor signaling pathway	6.61E-05	0.001812
101	positive regulation of phagocytosis	6.65E-05	0.001812
102	inflammatory response	7.36E-05	0.001986
103	neuron migration	8.37E-05	0.002216
104	sensory perception of sound	8.37E-05	0.002216
105	protein phosphorylation	9.08E-05	0.002382
106	learning or memory	0.0001	0.002606
107	negative regulation of cysteine-type endopeptidase activity involved in apoptotic process	0.000103	0.002652
108	embryo development	0.000105	0.002655
109	neuron differentiation	0.000105	0.002655
110	digestive tract development	0.000115	0.002833
111	negative regulation of smooth muscle cell proliferation	0.000115	0.002833
112	regulation of sensory perception of pain	0.000115	0.002833

Rank	GO Name	p-value	FDR
113	artery morphogenesis	0.000136	0.003273
114	regulation of cell differentiation	0.000137	0.003273
115	cardiac muscle contraction	0.000137	0.003273
116	DNA replication	0.000143	0.003339
117	cell surface receptor signaling pathway	0.000143	0.003339
118	response to wounding	0.000143	0.003339
119	heart contraction	0.000148	0.00342
120	response to activity	0.00015	0.003433
121	embryo implantation	0.000161	0.003613
122	pattern recognition receptor signaling pathway	0.000163	0.003613
123	smooth muscle cell migration	0.000163	0.003613
124	response to methotrexate	0.000163	0.003613
125	leukocyte cell-cell adhesion	0.000171	0.003766
126	negative regulation of protein kinase activity	0.000192	0.004203
127	axon guidance	0.000202	0.004376
128	protein autophosphorylation	0.000205	0.004406
129	morphogenesis of embryonic epithelium	0.00021	0.004429
130	regulation of ERK1 and ERK2 cascade	0.00021	0.004429
131	behavioral fear response	0.000212	0.004429
132	cell chemotaxis	0.000212	0.004429
133	cellular response to mechanical stimulus	0.000216	0.004466
134	adult walking behavior	0.000221	0.004536
135	peptidyl-tyrosine dephosphorylation	0.000256	0.00523
136	regulation of neuronal synaptic plasticity	0.000261	0.005269
137	cellular calcium ion homeostasis	0.000262	0.005269
138	negative regulation of neuron apoptotic process	0.000271	0.005398
139	immune response	0.000293	0.005806
140	positive regulation of cell adhesion	0.000297	0.005831
141	response to stress	0.000311	0.006073
142	osteoclast differentiation	0.000318	0.006161
143	intramembranous ossification	0.000321	0.006171
144	response to organic substance	0.000331	0.006329
145	positive regulation of peptidyl-tyrosine phosphorylation	0.00038	0.007212
146	positive regulation of reactive oxygen species metabolic process	0.000383	0.007223
147	positive regulation of peptidyl-threonine phosphorylation	0.000388	0.007223
148	positive regulation of bone resorption	0.000388	0.007223
149	skeletal system development	0.000415	0.00757
150	intracellular protein kinase cascade	0.000415	0.00757
151	glucose homeostasis	0.000415	0.00757
152	response to oxidative stress	0.0005	0.00899
153	response to nutrient	0.0005	0.00899
154	positive regulation of GTPase activity	0.000509	0.00899
155	circadian regulation of gene expression	0.000509	0.00899

Rank	GO Name	p-value	FDR
156	retinoic acid metabolic process	0.000509	0.00899
157	positive regulation of smooth muscle cell proliferation	0.000524	0.009131
158	protein stabilization	0.000524	0.009131
159	response to insulin stimulus	0.000538	0.009274
160	pachytene	0.000552	0.009274
161	negative regulation of ATPase activity	0.000552	0.009274
162	negative regulation of striated muscle tissue development	0.000552	0.009274
163	regulation of branching involved in prostate gland morphogenesis	0.000552	0.009274
164	cellular response to lipoteichoic acid	0.000552	0.009274
165	myelination	0.000577	0.009632
166	vasculogenesis	0.000639	0.010485
167	phagocytosis	0.000644	0.010485
168	positive regulation of mitosis	0.000644	0.010485
169	cellular response to calcium ion	0.000644	0.010485
170	negative regulation of cell growth	0.000649	0.010491
171	inactivation of MAPK activity	0.000655	0.010491
172	regulation of muscle contraction	0.000655	0.010491
173	skin development	0.000735	0.011701
174	response to hyperoxia	0.000755	0.011872
175	face morphogenesis	0.000755	0.011872
176	cellular response to hypoxia	0.000774	0.012101
177	regulation of cell growth	0.000826	0.012273
178	positive regulation of protein kinase activity	0.000826	0.012273
179	wound healing involved in inflammatory response	0.000827	0.012273
180	B cell proliferation involved in immune response	0.000827	0.012273
181	phosphatidylinositol catabolic process	0.000827	0.012273
182	collagen-activated tyrosine kinase receptor signaling pathway	0.000827	0.012273
183	embryonic camera-type eye formation	0.000827	0.012273
184	smooth muscle cell-matrix adhesion	0.000827	0.012273
185	sodium ion export	0.000827	0.012273
186	digestive tract morphogenesis	0.000829	0.012273
187	response to lipopolysaccharide	0.00085	0.012519
188	positive regulation of intracellular protein kinase cascade	0.000871	0.012611
189	T-helper 1 type immune response	0.000871	0.012611
190	female pregnancy	0.000872	0.012611
191	embryonic hindlimb morphogenesis	0.00088	0.012611
192	eating behavior	0.00088	0.012611
193	skeletal muscle cell differentiation	0.000925	0.013128
194	positive regulation of canonical Wnt receptor signaling pathway	0.000925	0.013128
195	activation of MAPK activity	0.00093	0.013128
196	response to nutrient levels	0.001017	0.014031
197	inner ear morphogenesis	0.001017	0.014031
198	amino acid transmembrane transport	0.001019	0.014031

Rank	GO Name	p-value	FDR
199	response to bacterium	0.001019	0.014031
200	neutrophil chemotaxis	0.001019	0.014031
201	positive regulation of activated T cell proliferation	0.001033	0.014085
202	negative regulation of phosphorylation	0.001033	0.014085
203	positive regulation of protein phosphorylation	0.00111	0.015051
204	chromosome segregation	0.001151	0.015536
205	liver development	0.001183	0.015883
206	response to cAMP	0.00121	0.016168
207	regulation of cell proliferation	0.001232	0.016168
208	positive regulation of neuroblast proliferation	0.001271	0.016168
209	response to light stimulus	0.001271	0.016168
210	negative regulation of I-kappaB kinase/NF-kappaB cascade	0.001271	0.016168
211	cytosolic calcium ion homeostasis	0.001271	0.016168
212	positive regulation of synaptic transmission, glutamatergic	0.001271	0.016168
213	negative regulation of angiogenesis	0.001279	0.016168
214	activation of innate immune response	0.001286	0.016168
215	response to molecule of bacterial origin	0.001286	0.016168
216	response to aluminum ion	0.001286	0.016168
217	vesicle organization	0.001286	0.016168
218	regulation of cell adhesion mediated by integrin	0.001286	0.016168
219	mesenchymal cell differentiation	0.001286	0.016168
220	organ morphogenesis	0.001318	0.016487
221	response to amphetamine	0.001348	0.016497
222	positive regulation of mesenchymal cell proliferation	0.001348	0.016497
223	positive regulation of interferon-gamma production	0.001348	0.016497
224	positive regulation of cell division	0.001348	0.016497
225	regulation of calcium ion transport	0.001348	0.016497
226	cytokinesis	0.001418	0.017197
227	fat cell differentiation	0.001418	0.017197
228	transcription from RNA polymerase II promoter	0.001425	0.017207
229	negative regulation of cell migration	0.001432	0.017215
230	sensory perception of smell	0.00154	0.018334
231	response to reactive oxygen species	0.001545	0.018334
232	cellular response to fibroblast growth factor stimulus	0.001545	0.018334
233	positive regulation of neuron differentiation	0.001685	0.019904
234	regulation of growth	0.001751	0.020598
235	male gonad development	0.001788	0.020757
236	glial cell migration	0.00181	0.020757
237	cerebral cortex radially oriented cell migration	0.00181	0.020757
238	positive regulation of tumor necrosis factor biosynthetic process	0.00181	0.020757
239	positive regulation of G-protein coupled receptor protein signaling pathway	0.00181	0.020757
240	centrosome duplication	0.00181	0.020757
241	regulation of pH	0.001858	0.021139

Rank	GO Name	p-value	FDR
242	vascular endothelial growth factor receptor signaling pathway	0.001858	0.021139
243	metabolic process	0.001899	0.021404
244	protein complex assembly	0.001905	0.021404
245	negative regulation of neuron differentiation	0.001905	0.021404
246	response to radiation	0.001983	0.022098
247	positive regulation of cell differentiation	0.001983	0.022098
248	blood vessel development	0.002093	0.023138
249	embryonic limb morphogenesis	0.002093	0.023138
250	regulation of action potential in neuron	0.002213	0.023839
251	positive regulation of phosphatidylinositol 3-kinase activity	0.002213	0.023839
252	cellular response to peptide hormone stimulus	0.002213	0.023839
253	negative regulation of canonical Wnt receptor signaling pathway	0.002294	0.023839
254	camera-type eye development	0.002295	0.023839
255	elevation of cytosolic calcium ion concentration	0.002405	0.023839
256	positive regulation of type IIa hypersensitivity	0.002447	0.023839
257	neutrophil activation involved in immune response	0.002447	0.023839
258	antigen processing and presentation of exogenous protein antigen via MHC class Ib, TAP-dependent	0.002447	0.023839
259	platelet degranulation	0.002447	0.023839
260	positive regulation of adaptive immune response	0.002447	0.023839
261	negative regulation of acute inflammatory response to antigenic stimulus	0.002447	0.023839
262	negative regulation of humoral immune response mediated by circulating immunoglobulin	0.002447	0.023839
263	negative regulation of Wnt receptor signaling pathway involved in heart development	0.002447	0.023839
264	detection of lipopolysaccharide	0.002447	0.023839
265	response to carbon monoxide	0.002447	0.023839
266	negative regulation of circadian sleep/wake cycle, sleep	0.002447	0.023839
267	regulation of retinal cell programmed cell death	0.002447	0.023839
268	Tie signaling pathway	0.002447	0.023839
269	monocyte aggregation	0.002447	0.023839
270	negative regulation of monocyte chemotactic protein-1 production	0.002447	0.023839
271	mesenchymal cell proliferation involved in ureteric bud development	0.002447	0.023839
272	metanephric glomerular capillary formation	0.002447	0.023839
273	negative regulation of pancreatic juice secretion	0.002447	0.023839
274	positive regulation of somatostatin secretion	0.002447	0.023839
275	chronic inflammatory response	0.002451	0.023839
276	generation of precursor metabolites and energy	0.002451	0.023839
277	positive regulation of fibroblast migration	0.002451	0.023839
278	cerebral cortex cell migration	0.002451	0.023839
279	actin filament-based movement	0.002451	0.023839
280	melanosome organization	0.002451	0.023839
281	regulation of GTPase activity	0.002451	0.023839

Rank	GO Name	p-value	FDR
282	membrane hyperpolarization	0.002451	0.023839
283	negative regulation of protein serine/threonine kinase activity	0.002451	0.023839
284	regulation of synaptic plasticity	0.002514	0.024366
285	oligodendrocyte differentiation	0.002614	0.025246
286	positive regulation of interleukin-6 production	0.002815	0.027005
287	positive regulation of inflammatory response	0.002815	0.027005
288	negative regulation of endothelial cell proliferation	0.003062	0.029065
289	lamellipodium assembly	0.003062	0.029065
290	negative regulation of fibroblast proliferation	0.003062	0.029065
291	ion transport	0.003143	0.02963
292	positive regulation of insulin secretion	0.003143	0.02963
293	anterior/posterior pattern specification	0.003158	0.02963
294	retina layer formation	0.003218	0.02963
295	positive regulation of urine volume	0.003218	0.02963
296	retinal metabolic process	0.003218	0.02963
297	apoptotic cell clearance	0.003218	0.02963
298	positive regulation of membrane protein ectodomain proteolysis	0.003218	0.02963
299	interleukin-1-mediated signaling pathway	0.003218	0.02963
300	palate development	0.003254	0.029865
301	blood coagulation	0.003536	0.032341
302	regulation of heart rate	0.003561	0.03235
303	lipopolysaccharide-mediated signaling pathway	0.003561	0.03235
304	negative regulation of endopeptidase activity	0.003836	0.034574
305	branching involved in ureteric bud morphogenesis	0.003881	0.034574
306	heart morphogenesis	0.003881	0.034574
307	learning	0.003881	0.034574
308	epithelial cell differentiation	0.003881	0.034574
309	cellular response to hormone stimulus	0.003881	0.034574
310	meiosis	0.004113	0.036127
311	cell migration involved in sprouting angiogenesis	0.004121	0.036127
312	immunoglobulin mediated immune response	0.004121	0.036127
313	prepulse inhibition	0.004121	0.036127
314	branching involved in salivary gland morphogenesis	0.004121	0.036127
315	positive regulation of protein kinase B signaling cascade	0.004154	0.036307
316	ureteric bud development	0.004294	0.037287
317	positive regulation of phosphatidylinositol 3-kinase cascade	0.004294	0.037287
318	associative learning	0.004722	0.038191
319	positive regulation of endothelial cell migration	0.004722	0.038191
320	adrenal gland development	0.004722	0.038191
321	negative regulation of tumor necrosis factor production	0.004722	0.038191
322	cellular response to drug	0.004738	0.038191
323	renal water homeostasis	0.004827	0.038191
324	axon target recognition	0.004827	0.038191

Rank	GO Name	p-value	FDR
325	cyclin catabolic process	0.004827	0.038191
326	regulation of smooth muscle cell migration	0.004827	0.038191
327	corpus callosum morphogenesis	0.004827	0.038191
328	anterior commissure morphogenesis	0.004827	0.038191
329	negative regulation of aldosterone biosynthetic process	0.004827	0.038191
330	negative regulation of lipid transport	0.004827	0.038191
331	interleukin-1 beta production	0.004827	0.038191
332	endothelial cell chemotaxis	0.004827	0.038191
333	positive regulation of endothelial cell chemotaxis by VEGF-activated vascular endothelial growth factor receptor signaling pathway	0.004827	0.038191
334	ERBB signaling pathway	0.004827	0.038191
335	regulation of cAMP-mediated signaling	0.004827	0.038191
336	meiotic chromosome segregation	0.004827	0.038191
337	negative regulation of endothelial cell differentiation	0.004827	0.038191
338	glucose import	0.004827	0.038191
339	branching morphogenesis of a nerve	0.004827	0.038191
340	mesenchyme development	0.004827	0.038191
341	endodermal digestive tract morphogenesis	0.004827	0.038191
342	cellular response to heparin	0.004827	0.038191
343	ductus arteriosus closure	0.004827	0.038191
344	positive regulation of long-term synaptic potentiation	0.004827	0.038191
345	calcium ion export	0.004827	0.038191
346	negative regulation of cortisol biosynthetic process	0.004827	0.038191
347	circadian rhythm	0.004851	0.038191
348	sensory perception of pain	0.004851	0.038191
349	positive regulation of cell growth	0.004851	0.038191
350	protein homooligomerization	0.004873	0.038191
351	axonogenesis	0.004883	0.038191
352	cell-cell adhesion	0.004883	0.038191
353	synaptic transmission, dopaminergic	0.005165	0.039611
354	phagocytosis, engulfment	0.005165	0.039611
355	antigen processing and presentation of exogenous peptide antigen via MHC class II	0.005165	0.039611
356	cell proliferation in forebrain	0.005165	0.039611
357	embryonic digestive tract development	0.005165	0.039611
358	negative regulation of peptidyl-tyrosine phosphorylation	0.005165	0.039611
359	heart trabecula formation	0.005165	0.039611
360	ATP catabolic process	0.005199	0.039758
361	Wnt receptor signaling pathway	0.005253	0.04006
362	peripheral nervous system development	0.00539	0.040768
363	phosphatidylinositol-mediated signaling	0.00539	0.040768
364	negative regulation of ERK1 and ERK2 cascade	0.00539	0.040768
365	DNA repair	0.005438	0.041013

Rank	GO Name	p-value	FDR
366	BMP signaling pathway	0.005724	0.04294
367	neuromuscular process controlling balance	0.005724	0.04294
368	Ras protein signal transduction	0.006121	0.045418
369	feeding behavior	0.006121	0.045418
370	bone mineralization	0.006121	0.045418
371	regulation of long-term neuronal synaptic plasticity	0.006121	0.045418
372	skeletal muscle contraction	0.00636	0.046196
373	complement activation	0.00636	0.046196
374	dopamine receptor signaling pathway	0.00636	0.046196
375	midgut development	0.00636	0.046196
376	regulation of ossification	0.00636	0.046196
377	regulation of bone mineralization	0.00636	0.046196
378	positive regulation of cytokinesis	0.00636	0.046196
379	regulation of peptidyl-tyrosine phosphorylation	0.00636	0.046196
380	positive regulation of cell-substrate adhesion	0.006916	0.049451
381	proton transport	0.006916	0.049451
382	embryonic forelimb morphogenesis	0.006916	0.049451
383	negative regulation of insulin secretion	0.006916	0.049451
384	developmental growth	0.006916	0.049451
385	cellular response to glucocorticoid stimulus	0.006916	0.049451
386	cytoskeleton organization	0.006974	0.049738

Supplementary Table 6

Pathway enrichment analysis by 60 minutes of ischemia followed 3 days of reperfusion

Rank	Pathway Name	p-value	FDR
1	Proteoglycans in cancer	5.20178E-13	1.11318E-10
2	PI3K-Akt signaling pathway	1.47893E-09	1.43859E-07
3	Regulation of actin cytoskeleton	2.01672E-09	1.43859E-07
4	Focal adhesion	4.39956E-09	2.35376E-07
5	Phagosome	8.78608E-09	3.76044E-07
6	ECM-receptor interaction	4.80098E-08	1.63437E-06
7	Malaria	5.34608E-08	1.63437E-06
8	MAPK signaling pathway	6.7552E-08	1.80702E-06
9	Olfactory transduction	1.4351E-07	3.41234E-06
10	Transcriptional misregulation in cancer	3.56585E-07	7.63092E-06
11	Staphylococcus aureus infection	4.98764E-07	9.70323E-06
12	Pathways in cancer	8.29502E-07	1.47928E-05
13	DNA replication	1.30226E-06	2.14372E-05
14	Cell cycle	3.87122E-06	5.65844E-05
15	Complement and coagulation cascades	3.96619E-06	5.65844E-05
16	Lysosome	4.23317E-06	5.66186E-05
17	Calcium signaling pathway	4.97466E-06	6.26222E-05
18	Amoebiasis	5.84076E-06	6.75772E-05

Rank	Pathway Name	p-value	FDR
19	Osteoclast differentiation	5.99985E-06	6.75772E-05
20	Circadian entrainment	1.04247E-05	0.000111545
21	Hypertrophic cardiomyopathy (HCM)	1.64007E-05	0.000167131
22	Hematopoietic cell lineage	1.82513E-05	0.000177535
23	Dilated cardiomyopathy	3.04571E-05	0.000276798
24	Pertussis	3.10427E-05	0.000276798
25	Rheumatoid arthritis	3.35989E-05	0.000287607
26	HTLV-I infection	3.86458E-05	0.000318085
27	NF-kappa B signaling pathway	4.47509E-05	0.000354692
28	Metabolic pathways	5.59131E-05	0.000427336
29	Tuberculosis	6.16746E-05	0.000455116
30	Cocaine addiction	8.72333E-05	0.000622264
31	Natural killer cell mediated cytotoxicity	0.000116874	0.000806805
32	Amphetamine addiction	0.000159015	0.001063414
33	Dopaminergic synapse	0.000172573	0.001091948
34	Viral myocarditis	0.000173487	0.001091948
35	Leishmaniasis	0.000262185	0.001603074
36	Cytokine-cytokine receptor interaction	0.000293165	0.001742703
37	Legionellosis	0.000473083	0.00273621
38	Salmonella infection	0.000493998	0.00278199
39	Nicotinate and nicotinamide metabolism	0.000545471	0.002993098
40	TGF-beta signaling pathway	0.000584636	0.003127805
41	Purine metabolism	0.000655519	0.003421488
42	Neuroactive ligand-receptor interaction	0.00067952	0.003462318
43	Gap junction	0.000941041	0.004683321
44	Protein digestion and absorption	0.001014691	0.004935089
45	Oocyte meiosis	0.001109851	0.005277957
46	Leukocyte transendothelial migration	0.001514322	0.007044887
47	Adherens junction	0.001554229	0.007076701
48	Antigen processing and presentation	0.001787758	0.007903776
49	HIF-1 signaling pathway	0.001809743	0.007903776
50	B cell receptor signaling pathway	0.002294334	0.00944319
51	Bacterial invasion of epithelial cells	0.002294334	0.00944319
52	Glutathione metabolism	0.002294607	0.00944319
53	Chemokine signaling pathway	0.002451254	0.009897515
54	Chagas disease (American trypanosomiasis)	0.00315845	0.012516821
55	Progesterone-mediated oocyte maturation	0.003764709	0.014648141
56	Insulin secretion	0.004295906	0.01612849
57	Prostate cancer	0.004295906	0.01612849
58	Cholinergic synapse	0.004460754	0.016458646
59	Circadian rhythm	0.005390332	0.019551372
60	Cell adhesion molecules (CAMs)	0.006536729	0.023231009
61	Fc gamma R-mediated phagocytosis	0.006621923	0.023231009

Rank	Pathway Name	p-value	FDR
62	Adipocytokine signaling pathway	0.00747007	0.025176942
63	Gastric acid secretion	0.00747007	0.025176942
64	Neurotrophin signaling pathway	0.007529553	0.025176942
65	Melanogenesis	0.007870502	0.025912114
66	Fanconi anemia pathway	0.008129688	0.026359896
67	p53 signaling pathway	0.008540536	0.027139464
68	Alcoholism	0.008623755	0.027139464
69	Hippo signaling pathway	0.010915687	0.033854448
70	NOD-like receptor signaling pathway	0.011181974	0.034184891
71	Retinol metabolism	0.01170794	0.03528872
72	African trypanosomiasis	0.013187412	0.039195918
73	Systemic lupus erythematosus	0.013700061	0.040161823
74	Primary immunodeficiency	0.014505624	0.041948696
75	Epstein-Barr virus infection	0.016786923	0.047898688
76	Renin-angiotensin system	0.019368028	0.05435216
77	Glioma	0.019556618	0.05435216
78	ErbB signaling pathway	0.020481397	0.056021133
79	Viral carcinogenesis	0.020680698	0.056021133
80	Small cell lung cancer	0.021560152	0.057673406
81	Wnt signaling pathway	0.02223602	0.058449926
82	Porphyrin and chlorophyll metabolism	0.022396701	0.058449926
83	Pancreatic cancer	0.023577671	0.060790623
84	Morphine addiction	0.025036915	0.063784521
85	Drug metabolism - cytochrome P450	0.026278375	0.066159674
86	Caffeine metabolism	0.027068238	0.067355849
87	Herpes simplex infection	0.02879432	0.070827408
88	Melanoma	0.029764297	0.071444303
89	Pancreatic secretion	0.030260062	0.071444303
90	Sphingolipid metabolism	0.030380521	0.071444303
91	Mineral absorption	0.030380521	0.071444303
92	Arrhythmogenic right ventricular cardiomyopathy	0.035058214	0.081548455
93	Mucin type O-Glycan biosynthesis	0.037660304	0.086659194
94	Salivary secretion	0.043034879	0.097324502
95	Axon guidance	0.043204802	0.097324502
96	Pyrimidine metabolism	0.048407953	0.107909395
